# Over Expression of NANOS3 and DAZL in Human Embryonic Stem Cells

**DOI:** 10.1371/journal.pone.0165268

**Published:** 2016-10-21

**Authors:** Sarita Panula, Ahmed Reda, Jan-Bernd Stukenborg, Cyril Ramathal, Meena Sukhwani, Halima Albalushi, Daniel Edsgärd, Michiko Nakamura, Olle Söder, Kyle E. Orwig, Shinya Yamanaka, Renee A. Reijo Pera, Outi Hovatta

**Affiliations:** 1 Division of Obstetrics and Gynecology, Department of Clinical Sciences, Intervention and Technology, Karolinska Institutet and Karolinska University Hospital, Huddinge, SE-141 86, Stockholm, Sweden; 2 Pediatric Endocrinology Unit, Department of Women’s and Children’s Health, Karolinska Institutet and Karolinska University Hospital, SE-171 76, Stockholm, Sweden; 3 Department of Genetics and Department of Obstetrics and Gynecology, Institute for Stem Cell Biology and Regenerative Medicine, Center for Reproductive and Stem Cell Biology, Stanford University, Stanford, CA, 94305, United States of America; 4 Department of Obstetrics, Gynaecology, and Reproductive Sciences, University of Pittsburgh School of Medicine, Magee-Womens Research Institute, Pittsburgh, PA, 15213, United States of America; 5 Sultan Qaboos University, College of Medicine and Health Sciences, Muscat, Oman; 6 Department of Cell and Molecular Biology, Karolinska Institutet, SE-171 77, Stockholm, Sweden; 7 Center for iPS Cell Research and Application, Kyoto University, Kyoto, 606-8507, Japan; 8 Gladstone Institute of Cardiovascular Disease, San Francisco, CA, 94158, United States of America; 9 Department of Cell Biology and Neurosciences and Department of Chemistry and Biochemistry, Montana State University, Bozeman, MT, 59717, United States of America; University of Texas at Austin Dell Medical School, UNITED STATES

## Abstract

The mechanisms underlying human germ cell development are largely unknown, partly due to the scarcity of primordial germ cells and the inaccessibility of the human germline to genetic analysis. Human embryonic stem cells can differentiate to germ cells *in vitro* and can be genetically modified to study the genetic requirements for germ cell development. Here, we studied NANOS3 and DAZL, which have critical roles in germ cell development in several species, via their over expression in human embryonic stem cells using global transcriptional analysis, *in vitro* germ cell differentiation, and *in vivo* germ cell formation assay by xenotransplantation. We found that NANOS3 over expression prolonged pluripotency and delayed differentiation. In addition, we observed a possible connection of NANOS3 with inhibition of apoptosis. For DAZL, our results suggest a post-transcriptional regulation mechanism in hES cells. In addition, we found that DAZL suppressed the translation of *OCT4*, and affected the transcription of several genes associated with germ cells, cell cycle arrest, and cell migration. Furthermore, DAZL over expressed cells formed spermatogonia-like colonies in a rare instance upon xenotransplantation. These data can be used to further elucidate the role of NANOS3 and DAZL in germ cell development both *in vitro* and *in vivo*.

## Introduction

Germ cells are highly specialized cells that are responsible for the transmission of genetic information between generations and thus, crucial for the survival of a species. Our understanding of human germ cell development is limited, and insights about the cellular and molecular mechanisms controlling the human germ cell lineage has in most parts been extrapolated from studies with model organisms. In order to recapitulate the human germ cell formation *in vitro*, human pluripotent stem (hPS) cells offer a promising strategy. Human embryonic stem (hES) cells and induced pluripotent stem (iPS) cells can differentiate into early germ cells and even into haploid spermatogenic cells *in vitro*, albeit with low efficiency [1–7]. In addition, hPS cells enable studying the genetic requirements for human germ cell development through genetic modification experiments.

The germ cell lineage is enriched with RNA-binding proteins (RBPs) and several of them have been identified to contribute to the differentiation and maintenance of germ cells in diverse model organisms [8]. The *NANOS* gene family of RBPs is highly conserved and localized to the germ cells among several species [9]. First discovered in *Drosophila*, the single *nanos* gene maintains the germ cell population by preventing further differentiation, apoptosis and somatic cell fate [10,11]. In mice, *Nanos2* is required for male fertility, while *Nanos3* has an earlier role in PGC development before sex determination [12]. *Nanos3* is expressed in PGCs after their specification until shortly after their arrival in the gonads, and it has been shown to maintain the PGC population during migration via suppression of apoptosis [12–14]. In human, *NANOS3* was recently shown to be expressed in early PGCs at 4 weeks of development with declining expression after 9 weeks of development [15,16]. The expression of *NANOS3* in human PGCs seems to be largely coupled with the expression of other early germ cell markers, Octamer-binding transcription factor 4 (*OCT4*, also known as *POU5F1*) and PR domain containing 1, with ZNF domain (*PRDM1)* [4]. In addition, knockdown of NANOS3 in hES cells results in decreased expression of pluripotency and germ cell–related genes upon differentiation [17], suggesting that NANOS3 may have a role in both self-renewal and maintenance of early germ cells in human.

Deleted in azoospermia-like (DAZL) is another germ cell specific RBP, which is important in multiple stages of germ cell development of both males and females in different species [18]. In mice, disruption of *Dazl* in fetal male germ cells results in failed mitotic arrest and continued expression of pluripotency markers [19]. In addition, over expression of *Dazl* in mouse ESCs inhibited the translation of pluripotency related factors SRY (sex determining region Y)-box 2 (*Sox2)* and Sal-like 4 (*Sall4)* [20,21]. *Dazl* acts also as a meiosis-promoting factor in mouse germ cells [22]. In human, *DAZL* is expressed in post-migratory germ cells after 7 weeks of development [15,16]. DAZL protein is first found in the nucleus, but paralleled with down regulation of OCT4 or initiation of meiosis, DAZL is relocated to the cytoplasm [23,24]. We have previously shown that over expression of DAZL in hPS cells induces meiotic initiation [1,2,25], indicating that DAZL has a meiosis-promoting role also in human germ cell development.

Here, we studied the effect of NANOS3 and DAZL over expression in hES cells, by global transcriptional analysis using mRNA sequencing, *in vitro* differentiation, and by *in vivo* germ cell formation assay by transplantation into the seminiferous tubules of germ cell-depleted immunodeficient mice.

## Methods

### Ethics approval

Approval for the use of human cells and tissue samples (Dnr 2014-267-31-4 (adult testis tissue) and Dnr 2013-457-31-4 (fetal testis tissue)) was obtained from the Ethics Board of Karolinska Institutet and the Regional Ethics Board in Stockholm. All patients gave written informed consent for donating samples and studies were performed according to the amended Declaration of Helsinki. The hES cell line HS401 (XY) was derived earlier at Karolinska Institutet (Karolinska University Hospital Huddinge, Stockholm, Sweden) (Dnr 454/02) [26]. Animal experiments included in the study have been approved by the Institutional Animal Care and Use Committees of Magee-Womens Research Institute and the University of Pittsburgh School of Medicine in accordance with the National Institutes of Health guidelines for the care and use of animals (assurance A3654-01).

### Transfection

hES cells were co-transfected with *piggyBac* transposon (2.5 μg) and transposase vector (2.5 μg) in a 6-well format using 5 μl PLUS reagent and 10 μl Lipofectamine LTX (Life Technologies) according to the manufacturer’s instructions. Two days after the transfection, cells were selected with 1 μg/ml puromycin (Life Technologies) for 6 days.

### Cell culture

The hES cell line HS401 (46, XY) [27] was cultured on hES cell-qualified Matrigel (Corning) -coated plates using mTeSR1 medium (StemCell Technologies). The cells were passaged using Accutase (Life Technologies), followed by an overnight incubation with 5 μM Y-27632 (Millipore).

Soriano ES Feeder cell line SNL 76/7 STO (Mutant Mouse Regional Resource Center) was cultured using 10% fetal bovine serum, 50 U/ml and 50 mg/ml penicillin-streptomycin in DMEM–Glutamax, and passaged using 0.05% Trypsin (all from Life Technologies). For feeder cell preparation, the cells were γ-irradiated with 100 Gy and plated on 0.1% gelatine (Sigma) coated plates at a density of 52,000 cells/cm^2^.

### *In vitro* differentiation

Colonies of HS401 cells were collected by incubation with 1 mg/ml Collagenase IV (Worthington Biochemical Corporation) for 40 min in 37°C and plated as small clumps on SNL feeders in 1:3 ratio using mTeSR1 medium. The next day, medium was changed to SSC-differentiation medium [5] consisting of basal medium α-MEM (Life Technologies), 0.2% w/v bovine serum albumin (BSA, Sigma), 1x Glutamax (Life Technologies), 10 mM HEPES (Sigma), 50 U/ml and 50 mg/ml penicillin-streptomycin, 50 μM β-mercaptoethanol (Life Technologies), 5 μg/ml human recombinant insulin (Sigma), 10 μg/ml holo-transferrin (Sigma), 30 nM sodium selenite (Sigma), 60 μM putrescine (Sigma), 2.36 μM palmitic acid (Sigma), 0.21 μM palmitoleic acid (Sigma), 0.88 μM stearic acid (Sigma), 1.02 μM oleic acid (Sigma), 2.71 μM linoleic acid (Sigma), 0.43 μM linolenic acid (Sigma), 1 ng/ml human recombinant bFGF (R&D Systems) and 20 ng/ml recombinant human GDNF (R&D Systems). The SSC-differentiation medium was gassed with 90% N_2_, 5% CO_2,_ 5% O_2_ gas mixture for 30s before changed to cells every two days.

### Gene expression analysis

RNA was extracted using RNeasy Mini kit with on-column DNase I digestion (Qiagen) according to the manufacturer’s instructions. cDNA was synthesized from 1 μg RNA with random hexamers and SuperScript III, followed by RNase H treatment (Life Technologies) according to the manufacturer’s instructions. Quantitative PCR reactions were run with the StepOnePlus Real-Time PCR System, using Taqman Universal PCR master mix and Taqman assays (all from Life Technologies). Relative quantity was calculated with 2^-ΔΔCt^ using the average value of housekeeping genes *GAPDH* and *RPLPO* for ΔCt.

mRNA sequencing and basic data processing was done by the National Genomics Infrastructure at SciLifeLab, Stockholm, Sweden. Briefly, strand-specific RNA libraries from 12 total RNA samples were sequenced on one lane on Illumina HiSeq2500 with a 2x100bp setup in HighOutput mode. Reads were mapped with Tophat/2.0.4 to the Human genome assembly GRCh37. Gene counts were generated using htseq/0.6.1 on bam files with duplicates included. FPKMs for genes and transcripts were generated using cufflinks/2.1.1 on bamfiles with duplicates included.

Differential gene expression analysis for mRNA sequencing data was done with edgeR using R. Batch effect of two samples (P1177_103 / *pbMOCK* 3 and P1177_109 / *pbNANOS3* 3) was used as a blocking variable in the analysis. Significance was accepted at FDR < 0.05.

### Immunostaining

Cells were fixed with 4% formaldehyde (Sigma) for 15 min and permeabilized with 0.3% Triton X-100 (Sigma) for 10 min. Cells were blocked with 5% donkey serum (Life Technologies), 1% BSA, and 0.1% Tween-20 (Sigma) in PBS (Life Technologies) for 1 h. Primary antibodies were diluted in 1% donkey serum, 0.1% BSA, and 0.1% Tween-20 in PBS and incubated overnight at 4°C. Cells were washed with 0.1% BSA and 0.1% Tween-20 in PBS and incubated with secondary antibodies in 1% donkey serum, 0.1% BSA, and 0.1% Tween-20 in PBS for 1h. DNA was counterstained with 1 μg/ml DAPI (Life Technologies) for 5 min.

Tissue sections were deparaffinized in Xylene (Sigma) and rehydrated through ethanol series (99.5%, 95% and 70%). Antigen retrieval was done in 0.01M sodium citrate buffer (pH 6.0) at 96°C for 30 min, followed by 30 min cooling at room temperature. Blocking was done with 3% H_2_O_2_ (Sigma) in MeOH (Sigma) for 30 min and with 20% chicken serum (Life Technologies) with 5% BSA in TBS for 30 min. Primary antibodies were diluted in serum blocking buffer and incubations were done in 4°C overnight. For human adult testicular tissue sections, fluorescence conjugated secondary antibodies were incubated in serum blocking buffer for 1h at room temperature. For xenografts, HRP-conjugated secondary antibody incubation was done in serum blocking buffer for 30 min at room temperature. Sections were treated with TSA Plus Fluorescein System (PerkinElmer) for 8 min. For double staining, second antigen retrieval was done as above and blocking was done with 3% H_2_O_2_ (Sigma) in TBS-T for 30 min and with 20% chicken serum (Life Technologies) with 5% BSA in TBS for 30 min. Primary and secondary antibody incubations were repeated as above and sections were treated with TSA Plus Cy3 System (PerkinElmer) for 8 min. Slides were mounted with Vectashield mounting medium with DAPI (Vector laboratories, USA).

Human second trimester fetal testis sample was obtained from Advanced Bioscience Resources (ABR Inc., Oakland, CA), fixed in formalin, and embedded in paraffin for cross-sectioning (AML Laboratories, USA). Human adult testicular tissue was obtained from the Department of Pathology, Karolinska Institutet, with informed consent and with the ethical permission from the regional ethics board (Dnr 2014-267-31-4), fixed in formaldehyde, and embedded in paraffin for cross-sectioning.

### Flow cytometry analysis

Cells were collected with 5 min incubation with Accutase at 37°C, centrifuged 300xg 5min and resuspended in PBS to 1x10^6^ cells/ml. Added 1 μl/ml of LIVE/DEAD Fixable Violet Dead cell Stain (Life Technologies) and incubated 30 min on ice. Washed cells with PBS and fixed with 4% formaldehyde 15 min at room temperature. Washed cells with FACS buffer (2% FBS in PBS) and permeabilized with 0.3% Triton X-100 10 min at room temperature. Washed cells with FACS buffer and incubated with 1:1000 rabbit a-DAZL (Abcam) antibody in FACS buffer 20 min on ice. Washed cells with FACS buffer and incubated with 1:200 donkey anti-rabbit Alexa Fluor 488 (Life Technologies) antibody in FACS buffer 20 min on ice. Washed with FACS buffer, passed cells through 35 μm cell strainer and analysed with BD LSRFortessa. Data was analysed using FlowJo v10 software.

### Western blotting

Protein was extracted from cells with 5 min incubation on ice with RIPA buffer (Sigma) and 1x Complete Mini Protease inhibitor cocktail (Roche). Protein concentration was measured with BCA Protein Assay kit (Pierce) and NanoDrop (ThermoScientific) according to the manufacturer’s instructions. Protein samples were denatured with 0.5M DTT (Life Technologies) in SDS sample buffer (2% SDS, 50mM Tris-HCl, and 10% glycerol) for 5 min on 95°C heat block. Denatured protein samples (20 μg) were run on 4–15% Criterion TGX gel (Bio-Rad) and transferred to Hybond-P membrane (Amersham) with semi-dry transfer method. Membrane was blocked with 5% non-fat milk (Santa Cruz antibodies) for 1 h and incubated with primary antibodies overnight at 4°C in blocking solution. Membrane was washed with 0.1% Tween-20 in TBS and incubated with HRP-conjugated secondary antibody in blocking solution for 1h. ECL Western Blotting Detection Reagents (Amersham) were used according to manufacturer’s instructions and membrane was imaged digitally using Fusion FX7 imaging system and Fusion software (Vilber Lourmat, France). For detecting the loading control GAPDH, membrane was stripped using 0.1 M β-mercaptoethanol and 2% SDS in 62.5 mM Tris-HCl for 45 min at 50°C, before re-starting the immunoblotting. Human adult testis protein medley (Clontech) was used as a positive control. Human fetal gonads were obtained from normal fetuses aborted legally in the first trimester with informed consent and with the ethical permission from the regional ethics board (Dnr 2013-457-31-4). Fetal gonads from 5 to 12 weeks of gestation were sonicated for 10 seconds in SDS sample buffer and kept on ice for 5 minutes. Lysate was then centrifuged at 13000 rpm at 4°C for 20 minutes and the supernatant was transferred to new tubes.

### Xenotransplantation assay

Xenotransplantation into the seminiferous tubules of busulfan-treated, immunodeficient mice (NCr nu/nu, Taconic) was done via cannulation of the efferent ducts with the approval of the University of Pittsburgh’s Institutional Animal Care and Use Committee, as previously described [28,29]. Briefly, mice were treated with a single dose of busulfan (40 mg/kg, Sigma) at six weeks of age. After five weeks or more, approximately 1*10^6^ cells in 7 μl were injected per testis. Six or seven mice with injections to both testes were used per cell line. Eight weeks after transplantation, testes were collected for whole mount staining or for paraffin embedding and cross sectioning.

For whole mount staining, the seminiferous tubules were dispersed with 1 mg/ml Collagenase IV and DNase I and fixed with 4% formaldehyde, as previously described [30]. Rabbit anti-primate testis cell primary antibody [30] and goat anti-rabbit IgG AlexaFluor 488 secondary antibody (Life Technologies) were used for detecting human cells.

For cross sectioning, testes were fixed in 4% formaldehyde over night at 4°C and dehydrated in 6-step ethanol series from 30% to 99.5% EtOH, and finally, in N-Butyl acetate (Sigma) for 24 h each. Testes were embedded in paraffin (Sigma) and sent to the Department of Laboratory Medicine at Karolinska Institutet, for serial cross sectioning and HE staining.

### Statistical analysis

For qPCR data, statistical analysis was done with one-way ANOVA and Tukey’s test for undifferentiated samples and with matched two-way ANOVA with Bonferroni’s test for differentiated samples, using Prism6 software (GraphPad). Statistical significance was accepted at p < 0.05. For DNA content analysis, testes weight and count of NuMA positive tubules, statistical analysis was done with one-way ANOVA by correcting for multiple testing with Bonferroni, using Prism6 software. Statistical significance was accepted at p < 0.05.

## Results

### Stable over expression of NANOS3 and DAZL in hES cells

**Fig 1:**
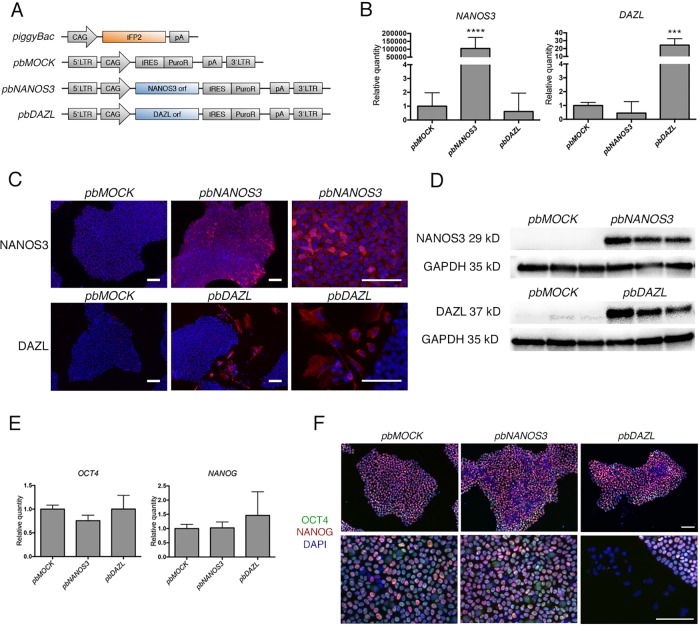
Stable over expression of NANOS3 and DAZL in hES cells. A) Schematic representation of *piggyBac* over expression constructs. Transposase expression vector (*piggyBac*) contained the IFP2 open reading frame (ORF) driven by the CAG promoter. Transposon expression vectors contained the ORF for *NANOS3* (*pbNANOS3*) or *DAZL* (*pbDAZL*) driven by the CAG promoter, followed by IRES element and puromycin resistance gene, and flanked by the *piggyBac* 5’ and 3’ long terminal repeats (LTRs). Vector without ORF was used as a control (*pbMOCK*). Human ES cells were co-transfected with the transposase vector and one of the transposon vectors, and selected with puromycin. B) Gene expression analysis of *NANOS3* and *DAZL* expression by qPCR. Samples from three separate transfections were used at passage 2 after transfection. Values are relative quantities normalized to *GAPDH* and *RPLPO*, and represented as mean ± SD. Statistical significance was tested by one-way ANOVA, **** p < 0.0001, *** p < 0.001. See also S1 Table. C) Immunofluorescence staining of NANOS3 (red) and DAZL (red) in *pbNANOS3* and *pbDAZL* cells, respectively. Note the DAZL positive cells outside the colonies. *pbMOCK* cells were used as negative control. Cells were counterstained with DAPI (blue). Scale bar 100 μm. D) Confirmation of NANOS3 and DAZL protein expression in *pbNANOS3* and *pbDAZL* cells by Western blotting. Samples at passage 2, 5 and 6 after transfection were used for each cell line. *pbMOCK* cells were used as negative control, and GAPDH was used as endogenous control for protein loading. See also S1B Fig. E) Gene expression of pluripotency markers *OCT4* and *NANOG* in transfected cells. Samples from three separate transfections were used at passage 2 after transfection. Values are relative quantities normalized to *GAPDH* and *RPLPO*, and represented as mean ± SD. See also S1 Table. F) Immunofluorescence staining of OCT4 (green) and NANOG (red). Note the negative *pbDAZL* cells outside the colonies. Cells were counterstained with DAPI (blue). Scale bar: 100 μm. See also S1C Fig.

To study the effect of germ cell specific genes *NANOS3* and *DAZL* in hES cells, we created over expression constructs containing the open reading frame (ORF) of the respective gene, driven by the CAG promoter (Fig 1A). To obtain stable transfection, we connected puromycin resistance gene by IRES element to the ORF and flanked them with *piggyBac* long terminal repeats (LTR) for *piggyBac* transposon mediated genomic integration [31]. We transfected hES cells, HS401 (46,XY) [27], with the *piggyBac* transposon vector and one of the transposase vectors using lipofection, and selected stable cell lines (*pbMOCK*, *pbNANOS3*, and *pbDAZL*) by puromycin treatment. Transfection was repeated three times for each transposase vector and the level of over expression was assessed by qPCR. Significantly higher expression of *NANOS3* and *DAZL* was confirmed for *pbNANOS3* (p < 0.0001) and *pbDAZL* cells (p < 0.001), respectively, relative to the *pbMOCK* control cells (Fig 1B, S1 Table). No effect was seen in *NANOS3* expression for *pbDAZL* cells. However, we did observe lower *DAZL* expression in *pbNANOS3* cells relative to *pbMOCK* cells, although this was not found statistically significant.

To test the protein translation of the transgenes, we analysed the cells by immunocytochemistry. *pbNANOS3* cells were positive for cytoplasmic expression of NANOS3, while *pbMOCK* cells were negative (Fig 1C). The *pbNANOS3* cells grew in colonies, with morphology of undifferentiated hES cells, similar to *pbMOCK* cells (Fig 1C). For *pbDAZL* cultures, we observed cells outside the colonies that stained positive for DAZL in the cytoplasm, while majority of the cells in the colonies were negative, indicating there might be additional post-transcriptional regulation for the translation of *DAZL* in hES cells. Indeed, *DAZL* is normally expressed in undifferentiated hES cells, but not translated to protein [1,32]. To quantify the number of DAZL expressing cells, we performed flow cytometry analysis and found that 2.8% of *pbDAZL* cells expressed DAZL protein (S1A Fig).

To confirm the protein expression of the transgenes and to test the stable transfection of the cells, we performed Western blot analysis with samples from passage 2, 5 and 6 after transfection for all cell lines (Fig 1D). Protein bands for NANOS3 and DAZL were observed in all passage numbers for *pbNANOS3* and *pbDAZL* samples, respectively, confirming the stable transfection of the cells. The level of DAZL expression in *pbDAZL* cells was similar to adult testis sample (S1B Fig). NANOS3 expression was not detected in fetal or adult testis with this antibody (S1B Fig).

Given the similarities in colony morphology of the *pbNANOS3* and *pbDAZL* cells relative to *pbMOCK*, we analysed the expression of pluripotency markers *OCT4* and *NANOG*, and found no change in the expression levels by qPCR (Fig 1E, S1 Table). Immunocytochemistry also showed similar staining pattern of OCT4 and NANOG for *pbNANOS3* and *pbDAZL* cell colonies, relative to *pbMOCK* (Fig 1F). Notably, while *pbDAZL* cells in colonies were positive for OCT4, the DAZL positive cells observed outside the colonies were negative for OCT4 (S1C Fig).

### Differentially expressed genes in NANOS3 and DAZL over expressed cells identified by mRNA sequencing

**Fig 2:**
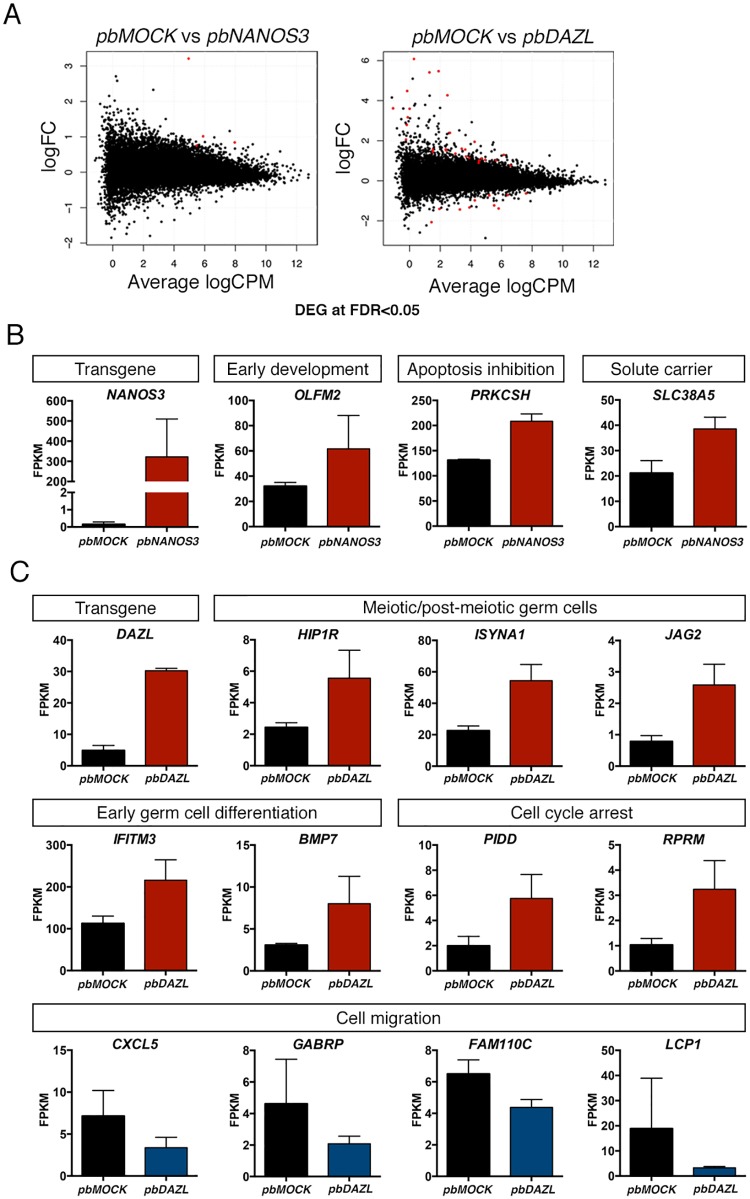
Differentially expressed genes in *pbNANOS3* and *pbDAZL* cells in undifferentiated culture conditions. A) Differential gene expression analysis for transfected cells by mRNA sequencing. Samples from three separate transfections were used at passage 2 after transfection. Results are shown in MA-plot with log fold change (logFC) values in the *pbNANOS3* or *pbDAZL* versus the *pbMOCK* sample, plotted against average log counts per million (logCPM). Significant genes (FDR < 0.05) are marked as red: *pbNANOS3* (4 genes), *pbDAZL* (38 genes). See also S2 Table. Selected differentially expressed genes for B) *pbNANOS3* and C) *pbDAZL*, with suggested functions. Values are Fragments Per Kilobase Of Exon Per Million Fragments (FPKM), and represented as mean ± SD. See also S1D and S2 Figs.

We next screened the cells for global transcriptional differences by mRNA sequencing and found that overall, both *pbNANOS3* and *pbDAZL* cells had very similar gene expression profiles relative to *pbMOCK* cells in undifferentiated culture conditions (Fig 2A). We performed differential gene expression analysis and found four genes up regulated in *pbNANOS3* cells relative to *pbMOCK*: *NANOS3*, Olfactomedin 2 *(OLFM2)*, Protein kinase C substrate 80K-H *(PRKCSH)*, and Solute carrier family 38 member 5 (*SLC38A5*) (Fig 2A and 2B, S2 Table). *OLFM2* belongs to the family of olfactomedin domain-containing proteins, which are associated in early development and cell differentiation [33]. *PRKCSH* encodes the beta-subunit of glucosidase II and has been shown to be associated with inhibition of apoptosis and induction of cell proliferation of lung cancer cells [34]. *SLC38A5* encodes a transporter for neutral amino acids. Surprisingly, no significantly down regulated genes were found in *pbNANOS3* cells. However, we observed that the higher the transgene expression of *NANOS3* was for *pbNANOS3* samples, the lower the expression of *DAZL* (S1D Fig).

In *pbDAZL* cells, we found 28 up regulated genes, including *DAZL*, and 10 down regulated genes, relative to *pbMOCK* cells (Fig 2A and 2C, S2 Table), even though only a subpopulation of *pbDAZL* cells expressed DAZL protein. Among the up regulated genes, Interferon-induced transmembrane protein 3 (*IFITM3)* and Bone morphogenetic protein 7 (*BMP7)* are associated with early germ cell development in mice [35,36]. In addition, three of the up regulated genes, Huntingtin interacting protein 1 related (*HIP1R*), Inositol-3-phosphate synthase 1 *(ISYNA1)*, and Jagged 2 *(JAG2)*, have a role in meiotic or post-meiotic germ cells. *HIP1R* has been shown to be required for differentiation and survival of post-meiotic spermatids in mice [37]. *ISYNA1* has a critical role in the *myo*-inositol biosynthesis pathway and has a high activity in the testes with expression in Sertoli cells, pachytene spermatocytes and round spermatids in mice [38]. *JAG2* is one of the several ligands that activate Notch receptor and it is expressed in the round and elongated spermatids of rat and human [39]. Two of the up regulated genes, p53-induced death domain protein 1 *(PIDD1)* and Reprimo *(RPRM)*, are associated with cell cycle arrest at G1 or G2 phase, respectively [40,41]. Notably, from the 10 down regulated genes in *pbDAZL* cells, 4 have been shown to play a role in cell migration: Chemokine (C-X-C motif) ligand 5 (*CXCL5)* [42], Gamma-aminobutyric acid A receptor, pi *(GABRP)* [43], Family with sequence similarity 110, member C (*FAM110C)* [44] and Lymphocyte cytosolic protein 1 (*LCP1)* [45].

To study whether the transcriptional changes in *pbNANOS3* and *pbDAZL* cells were reflected at a protein level, we performed immunocytochemistry with selected markers that are also expressed in the human testis according to the Human Protein Atlas. We first confirmed that all tested markers: OLFM2, PRKCSH, IFITM3, CXCL5, ISYNA1 and PIDD1, were expressed in normal human testis (S2A Fig). We found that the staining intensity of PRKSCH was higher in *pbNANOS3* cells relative to *pbMOCK* cells (S2B Fig), confirming the over expression also at protein level. No expression of OLFM2 was detected for *pbMOCK* or *pbNANOS3* cells. For *pbDAZL* cells, we focused on DAZL positive cells outside the colonies, however, no up regulation of *IFITM3*, *ISYNA1* or *PIDD1* translation was observed for DAZL positive *pbDAZL* cells (S2B Fig). No expression of CXCL5 was detected for *pbMOCK* or *pbDAZL* cells.

### Delayed *in vitro* differentiation for *pbNANOS3* cells

We next set to analyse the effect of NANOS3 and DAZL over expression for *in vitro* germ cell differentiation. We chose a previously published method for directed differentiation of hES cells to germ cells on SNL-feeders using a spermatogonial stem cell medium supplemented with Glial cell line-derived neurotrophic factor (GDNF) and basic fibroblast growth factor (bFGF) [5]. The differentiation of *pbMOCK*, *pbNANOS3* and *pbDAZL* cells was repeated three times at consecutive passages (6–8), using cell lines established from one transfection, to control the variance resulting from the transgene expression levels.

**Fig 3:**
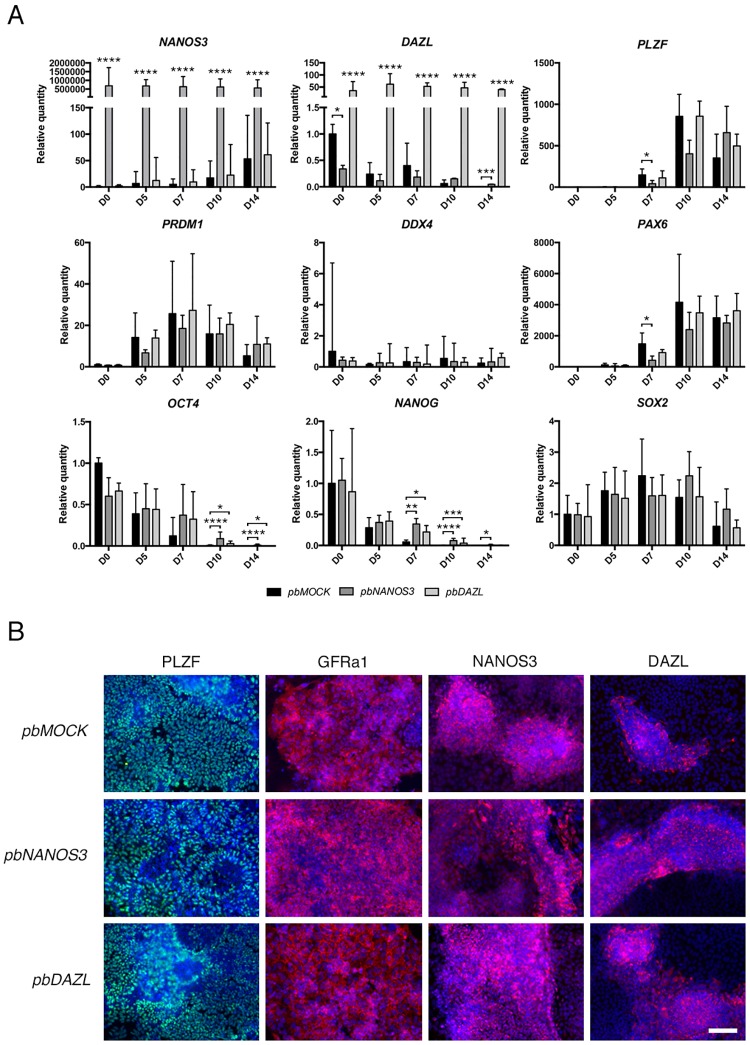
Analysis of *in vitro* differentiated *pbNANOS3* and *pbDAZL* cells. *A*) Gene expression analysis for cells differentiated on SNL-feeders using SSC medium for 5, 7, 10 or 14 days, and for cells in undifferentiated culture conditions (D0). Samples from three separate experiments were used at passage 6–8 after transfection. Values are relative quantities normalized to *GAPDH* and *RPLPO*, and represented as mean ± SD. Statistical significance was tested by two-way ANOVA, **** p < 0.0001, *** p < 0.001, ** p < 0.01, * p < 0.05. See also S3 Table. B) Immunofluorescence staining of transfected cells differentiated for 14 days on SNL-feeders; PLZF (green), GFRa1 (red), NANOS3 (red) and DAZL (red). Cells were counterstained with DAPI (blue). Scale bars: 100 μm. See also S3 Fig.

We collected undifferentiated cells (D0) and cells differentiated for 5, 7, 10 and 14 days for gene expression analysis (Fig 3A, S3 Table). The transgene expression of *NANOS3* and *DAZL* remained the same for *pbNANOS3* and *pbDAZL* cells, respectively, throughout the differentiation. The expression of *DAZL* was significantly lower for D0 *pbNANOS3* cells relative to *pbMOCK* (p < 0.05), when biological replicates from the same transfection experiment were used. We found that the expression of germ cell-related Promyelocytic leukaemia zinc finger (*PLZF)* (p < 0.0001) and *PRDM1* (p < 0.0001) were up regulated in *pbMOCK*, *pbNANOS3*, and *pbDAZL* cells, and *NANOS3* expression (p < 0.0001) was up regulated in *pbMOCK* and *pbDAZL* cells upon differentiation. We did not, however, observe any induction of *DDX4* in either of the cell lines. In addition, *DAZL* expression (p < 0.0001) was down regulated upon differentiation in *pbMOCK* and *pbNANOS3* cells. Instead, we observed induction of Paired box 6 (*PAX6)* (p < 0.0001), as well as maintained *SOX2* expression during the differentiation in all of the cell lines, suggesting a heterogeneous differentiation to neuronal cells as well as to early germ cells. Indeed, GDNF has been shown to induce neuronal differentiation from hES cells [46].

When comparing the differentiation between the cell lines, no difference in the expression of *PRDM1* was observed at any time point for *pbNANOS3* or *pbDAZL* cells, relative to *pbMOCK* cells, or in *NANOS3* expression for *pbDAZL* cells relative to *pbMOCK* cells. We did, however, observe a significantly lower expression of *PLZF* (p < 0.05) and *PAX6* (p < 0.05) for *pbNANOS3* cells at D7 relative to *pbMOCK* cells. Furthermore, we found that although the expression of *OCT4* and *NANOG* was down regulated upon differentiation in all cell lines (p < 0.0001) relative to D0, in *pbNANOS3* cells the expression of *OCT4* remained higher at D10 and D14 (p < 0.0001) and the expression of NANOG remained higher at D7, D10 and D14 (p < 0.01, p < 0.0001, p < 0.05, respectively) relative to *pbMOCK* cells. Also the expression of *DAZL* was higher (p < 0.0001) for *pbNANOS3* cells at D14 of differentiation relative to *pbMOCK* cells, which may result from remaining undifferentiated cells. Surprisingly, also in *pbDAZL* cells the expression of *OCT4* remained higher at D10 and D14 (p < 0.05) and the expression of *NANOG* remained higher at D7 and D10 (p < 0.05, p < 0.001) relative to *pbMOCK* cells.

Next, we analysed the protein expression of several markers for the transfected cells after 14 days of differentiation. Similar to previously published results [5], we found several colonies positive for PLZF in all differentiated cell lines and majority of the cells were positive for GDNF family receptor alpha 1 (GFRa1), a marker for spermatogonial stem cells [47] (Fig 3B), while undifferentiated hES cells were negative for both markers (S3A Fig). *pbNANOS3* cells were still positive for NANOS3 protein, and NANOS3 expression was also induced in several colonies of *pbMOCK* and *pbDAZL* cells after 14 days of differentiation (Fig 3B), supporting the qPCR data. In addition, expression of DAZL and PRDM1 was observed in some *pbMOCK*, *pbNANOS3* and *pbDAZL* cells after differentiation (Fig 3B, S3B Fig), further indicating the differentiation of early germ cells. We observed a similar DAZL expression in *pbMOCK* and *pbNANOS3* cells compared to *pbDAZL* cells after 14 days of differentiation, which may cause the effects of *DAZL* over expression to be undetected with this method.

As expected from the qPCR results, we did not find any positive cells for DDX4 in *pbMOCK*, *pbNANOS3* or *pbDAZL* differentiation cultures (S3B Fig), in contrary to previously published results with the same method [5]. In addition, few NANOG positive cells were observed for all cell lines even after 14 days of differentiation, indicating remaining undifferentiated cells (S3B Fig). Also, several SOX2 positive cells were observed for all cell lines, further suggesting a heterogeneous differentiation also towards the neuronal lineage (S3B Fig). The discrepancies of the differentiation results to the previously published work may be due to cell line differences, however, in our hands the method only supported the differentiation of PGC-like cells, and no evidence of later stage germ cells was observed.

### Colonies of spermatogonia-like cells from *pbDAZL* cells after xenotransplantation

**Fig 4:**
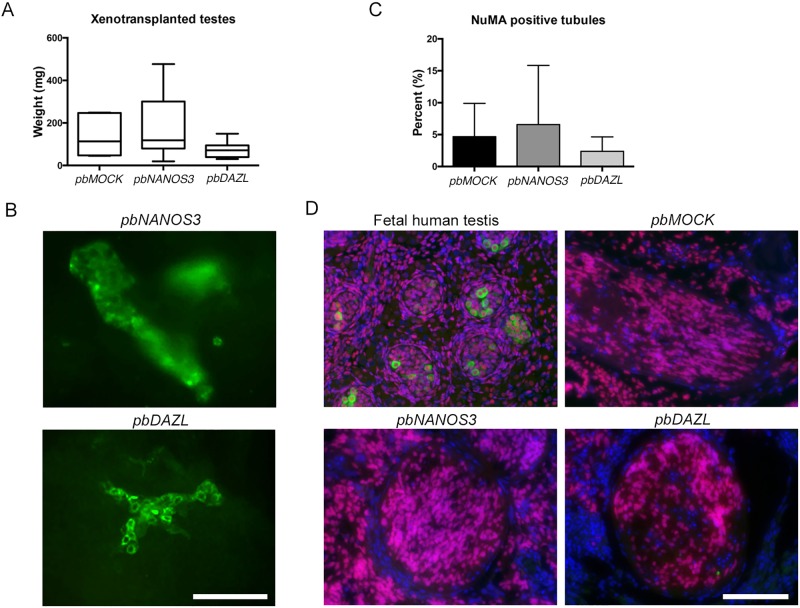
Xenotransplantation of transfected cells into the seminiferous tubules of mouse testes. A) Weight of the xenografts collected 8 weeks after transplantation, n ≥ 3. B) Whole mount staining with an antibody against primate testis cells. A cell clump was observed in one *pbNANOS3* transplanted testis. Six colonies with spermatogonial characteristics were observed in one *pbDAZL* transplanted testis, representative image shown. Scale bar: 100 μm. See also S4 Table. C) Serial sections from xenografts with weight < 150mg were screened for intratubular NuMA positive cells and percentage of positive tubules was calculated. Data are represented as mean ± SD, n ≥ 3. See also S4 Table. D) Immunofluorescence staining for human specific NuMA (red) and DDX4 (green). Human fetal testis sample was used as positive control. Representative images with intratubular NuMA positive and DDX4 negative cells are shown for *pbMOCK*, *pbNANOS3* and *pbDAZL* xenografts. Cells were counterstained with DAPI (blue). Scale bar: 100 μm.

To investigate the *in vivo* germ cell differentiation of *pbMOCK*, *pbNANOS3*, and *pbDAZL* cells, we used a previously described xenotransplantation assay [29,30]. Undifferentiated cells were injected via the efferent ducts into the seminiferous tubules of immunodeficient nude mice, which had been treated with 40 mg/kg busulfan to eliminate the endogenous mouse germ cells. For each cell line, 12–14 testes were injected. After up to two months, the testes were collected for either whole-mount staining to find possible chains of spermatogonia, or serial sectioning of fixed tissue for cell characterization by immunohistochemistry (S4 Table). The weight of the testis xenografts collected after 8 weeks, varied from 37 mg to 316 mg, with no significant difference between the sample groups (Fig 4A). Due to the large cell masses formed in most of the testes, whole-mount staining could only be performed for a small subgroup (S4 Table). Using an antibody specific to primate testis cells [30] we observed six human donor cell-derived colonies in one of the testes transplanted with *pbDAZL* cells (Fig 4B, representative colony shown). The cell colonies, ranging in size from 5 to 18 cells, had similar characteristic features described for spermatogonia: ovoid shape cells with high nuclear to cytoplasmic ratio and connected by intercytoplasmic bridges [30]. For *pbNANOS3* cells transplanted testis, a clump of human cells lacking the spermatogonial features was seen (Fig 4B), whereas no positive cells were found in the analysed *pbMOCK* transplanted testes.

For further analysis of the xenografts, six testes from each sample group were cross-sectioned and stained with hematoxylin and eosin. Majority of the testes had a dominating tumour component in the interstitial space and at least one testis in all sample groups had teratoma-like formation (S4 Table, S4A Fig), which was also observed in the previous reports using hES cells [28,48]. We observed several tubules containing extensive cell growth in all sample groups, indicative of donor cell origin (S4B Fig). Surprisingly, we also observed some tubules with teratoma-like differentiation in all sample groups, indicating disruption of the naïve niche of the tubule. In some xenografts, recovery of endogenous mouse spermatogenesis with spermatids was observed in the tubules (S4 Table, S4C Fig), which is sometimes expected for busulfan-treated mice after the 2-month transplantation period [49].

To confirm the colonization of the donor cells in the xenografts, we screened serial sections with a human specific antibody against Nuclear mitotic apparatus protein 1 (NuMA), which is expressed in the nuclei of most cell types [50], and with DDX4 antibody to quantify possible germ cell differentiation. Xenografts with weight less than 150 mg were included in the screening as they had a larger component of naïve testis structures left (*pbMOCK*: n = 4, *pbNANOS3*: n = 3, *pbDAZL*: n = 6). We counted the total number of tubules and tubules with NuMA positive cells and found a similar percentage (2.4–8.7%) of human donor cell colonization for all sample groups (Fig 4C), indicating a stable injection method and no preferential colonization or tubule rupture for any sample group. We observed that most of the NuMA positive tubules were full of donor cells, however, no double positive cells for DDX4/NuMA were found (Fig 4D). To analyse whether differentiation to early germ cells that do not yet express DDX4 could be observed, we stained the xenografts against DAZL, Developmental pluripotency associated 3 (DPPA3) and Undifferentiated embryonic cell transcription factor 1 (UTF1) antibodies, however, no double positive cells with NuMA could be detected. In most of the xenografts, we observed extensive tumour formation in the interstitial space and majority of the tubules with colonization were full of human donor cells that most likely disrupted the naïve niche of the tubules, which could not support the differentiation of either transfected donor cell line to germ cells.

## Discussion

The molecular mechanisms controlling human germ cell development, particularly in the embryonic stages, are poorly understood and often extrapolated from transgenic studies with model organisms. hES cells offer an accessible strategy to examine human germ cell development *in vitro*, especially the genetic requirements for germ cell development by over expressing or silencing of candidate genes. Here, we show the effects of NANOS3 and DAZL over expression in hES cells by focusing on early events before and after germ cell differentiation.

We found that over expression of NANOS3 did not interfere with the maintenance of undifferentiated hES cells (Fig 1), and upon *in vitro* differentiation, *pbNANOS3* cells had delayed down regulation of *OCT4* and *NANOG* expression and delayed up regulation of *PLZF* and *PAX6* expression (Fig 3A). It has been shown that knockdown of NANOS3 in differentiated hES cells results in decreased expression of pluripotency markers [17]. In addition, *NANOS3* expression is coupled with *OCT4* expression in early human germ cells [4]. Thus, our results support the previous finding that NANOS3 may have a role in the self-renewal and maintenance of early human germ cells [4,17].

Global transcriptional analysis confirmed the similarity of *pbNANOS3* cells to the control cells, and only three genes were found up regulated in the *pbNANOS3* cells (Fig 2). From these three genes, *PRKCSH* was confirmed to also have a higher protein expression level in *pbNANOS3* cells (S2 Fig). *PRKCSH* has been associated with protection from apoptosis and promoting self-renewal in lung cancer cell line [34]. In mice, *Nanos3* protects the PGC population during migration by suppressing apoptosis [13], thus it is possible that also human NANOS3 has this conserved role. However, further connection of *PRKCSH* to apoptosis and to NANOS3 would need to be established with future studies.

NANOS3 has been shown to interact with CCR4-NOT deadenylase complex, which represses translation and promotes mRNA degradation [51]. We did not, however, detect any down regulated gene expression in *pbNANOS3* cells with global transcriptional analysis (Fig 2). It is possible that hES cells in undifferentiated culture conditions do not have the mRNA targets of NANOS3 repression present and thus none were picked up in our analysis. However, we did observe a possible down regulation of *DAZL* mRNA in *pbNANOS3* cells (Figs 1B and 3A, S1D Fig). This seemed to be the case only with high level of *NANOS3* over expression (S1D Fig), suggesting that *DAZL* may be a low affinity target of NANOS3. Interestingly, mouse NANOS2 protein has been shown to associate with *Dazl* mRNA, triggering its degradation [52,53]. The interaction between NANOS3 and *DAZL* warrants further investigation.

We found that upon over expression of *DAZL* in hES cells, DAZL protein was expressed in the cytoplasm of cells that had a changed morphology and located outside the typical undifferentiated hES cell colonies. Although we did not detect down regulation of *OCT4* mRNA in the whole population of *pbDAZL* cells, we found that cells positive for DAZL protein were negative for OCT4 (S1C Fig), suggesting that DAZL represses the translation of *OCT4*. Indeed, cytoplasmic DAZL expression in human germ cells is observed around the time when OCT4 expression is lost [23,24]. Thus, it would be interesting to study whether *OCT4* mRNA is a direct target of DAZL or if the repression is an indirect downstream effect of DAZL expression. Surprisingly though, we observed a higher expression of *OCT4* and *NANOG* mRNA after *in vitro* differentiation in *pbDAZL* cells relative to *pbMOCK* cells (Fig 3A). However, only few positive cells for NANOG protein were observed after differentiation for *pbDAZL* cells, similar to *pbMOCK* cells, suggesting that the observed higher expression of *NANOG* is only at the mRNA level.

Over expression of *DAZL* in hES cells resulted only in a small subpopulation of cells expressing DAZL protein (2.8%, S1A Fig). Interestingly, *DAZL* transcripts are normally found in undifferentiated hES cells, without protein translation [1,32], indicating that hES cells may have a post-transcriptional regulation of *DAZL*, which is also affecting the exogenous *DAZL* transcripts. We have previously shown that over expression of *DAZL* induces meiosis in hPS cells, although with low numbers (4–15% of meiotic cells) [1,2,25]. It is possible that the low numbers are partly due to translational inhibition of *DAZL* in the transfected cells. Our stable over expression system could provide a useful platform to elucidate the mechanism for post-transcriptional regulation of *DAZL* in future studies.

We found several genes up or down regulated in *pbDAZL* cells by global transcriptional analysis, despite the small number of cells expressing DAZL protein (Fig 2, S2 Table). Two of the up regulated genes, *IFITM3* and *BMP7*, are associated with early germ cell differentiation [35,36], and three of the up regulated genes, *HIP1R*, *ISYNA1* and *JAG2*, are expressed in the meiotic or post-meiotic germ cells in mice or rat [37–39]. Interestingly, *HIP1R* has an important role in the accurate congression and segregation of chromosomes in mitosis [54], however, its function in meiosis has not yet been studied. Although we did not observe up regulation of the markers at protein level, their connection to DAZL and especially to meiotic initiation would be interesting to study further.

We found two up regulated genes associated with cell cycle arrest, *PIDD1* [40] and *RPRM* [41], in *pbDAZL* cells. In addition, we found that four out of the ten down regulated genes in *pbDAZL* cells have been associated with cell migration; *CXCL5* [42], *GABRP* [43], *FAM110C* [44], and *LCP1* [45]. In mice, DAZL has a role in promoting mitotic arrest in fetal male germ cells [19], however, no connection between DAZL and inhibition of cell migration has been made before. Although our results are merely suggestive and only observed at the gene expression level, they raise interest for future functional studies for the connection of DAZL to cell cycle arrest and cell migration.

Due to the small number of *pbDAZL* cells with DAZL protein expression, we expected to see clear differences in the DAZL expressing cells for the up and down regulated genes at a protein level by immunostaining. However, no up regulation of IFITM3, ISYNA1 or PIDD1, or down regulation of CXCL5 protein could be observed in DAZL positive cells (S2 Fig). We speculate that DAZL may affect the mRNA transcript levels of these genes, either directly or indirectly, but does not affect their translation. It is also possible that the high level of DAZL expression may have caused differential expression of mRNAs with low affinity recognition motifs that may not have physiological relevance.

We found that in one of the xenotransplanted testis, *pbDAZL* cells formed spermatogonia-like colonies in mouse seminiferous tubules (Fig 4B), similar to human SSCs transplanted into the murine testis as shown by Nagano *et al* 2002 [55]. We did not observe similar colony formation for *pbNANOS3* cells or for *pbMOCK* cells. Similarly, spermatogonia-like colony formation has not been observed in previous reports with hPS cell transplantation [28,56]. Unfortunately, only a small number of testes could be analysed for colony formation by whole mount staining because of the extensive tumour formation in majority of the xenografts, thus we cannot conclude that the colony formation would be specific for *pbDAZL* cells. In addition, due to the limited amount of material, further characterization of the cell colonies could not be performed. The low number of cells positive for DAZL protein in *pbDAZL* cells could explain the observed rare occasion of spermatogonia-like colony formation. It would be interesting to study the colony formation by transplanting only DAZL protein expressing cells, however, there are currently no known cell surface markers specific for DAZL expressing cells for efficient isolation of the cells.

In contrast to previous reports [28,56], we found no DDX4 positive donor cells in the seminiferous tubules by immunohistochemistry. This might be due to differences in hES cell lines, and because the naïve niche of the tubule was compromised due to the high number of colonized donor cells inside the tubule. Thus, germ cell differentiation could not be efficiently supported. Improvements for the xenotransplantation assay are needed in order to minimize the tumour formation when hPS cells are used, in particular, optimizing the injected cell number or using *in vitro* differentiated cells.

Studying germ cell development with hES and iPS cells offer several benefits. First, they offer an *in vitro* approach to study the genetic requirements for germ cell development. Second, they can be used to identify signalling pathways and growth factors that influence germ cell development. Third, they can be used to model infertility using patient specific iPS cells that may ultimately lead to new treatments for infertility. Improving the *in vitro* differentiation of germ cells would be essential for gaining more knowledge in all of these categories. Recently, progress has been made to achieve efficient differentiation of hES cells to PGC-like cells [6,7], however further differentiation steps still need to be developed. Our data provide new information and suggestions of NANOS3 and DAZL function in hES cells that may be used to optimize *in vitro* differentiation of germ cells from hES cells and to further elucidate the role of NANOS3 and DAZL in germ cell development *in vivo*.

## Supporting Information

S1 Fig Analysis of undifferentiated *pbMOCK*, *pbNANOS3* and *pbDAZL* cells. **A)** Flow cytometry analysis for quantification of DAZL positive cells in *pbDAZL* cultures. 2.8% of *pbDAZL* cells expressed DAZL protein. *pbMOCK* cells were used as negative control. **B)** Assessment of protein levels by Western blotting in over expressed cells relative to normal material obtained from the first trimester human fetal and adult testis samples. DAZL expression in *pbDAZL* cells was similar to adult testis sample, whereas no NANOS3 protein could be detected in either fetal or adult testis samples with this antibody. **C)** Immunofluorescence staining for OCT4 (green) and DAZL (red) to confirm lack of OCT4 expression in DAZL positive cells found outside the colonies. Scale bar indicates 100 μm. **D)** Gene expression analysis by mRNA sequencing. Expression of *NANOS3* and *DAZL* are shown for biological replicates of *pbMOCK* and *pbNANOS3* cells. The expression of *DAZL* is lower in *pbNANOS3* samples with highest *NANOS3* expression. Values are Fragments Per Kilobase Of Exon Per Million Fragments (FPKM).
(TIF)

S2 Fig Protein expression of selected markers found to be differentially expressed in *pbNANOS3* or *pbDAZL* cells relative to *pbMOCK* by mRNA sequencing. **A)** Human adult testis sections were immunostained for the selected markers (green) as a positive control. Arrowhead indicates Leydig cells and # indicates seminiferous tubular cells. Scale bar for the selected markers indicates 20 μm and for IgG controls 50 μm. **B)** Immunofluorescence staining of selected markers (green) together with NANOS3 (red) or DAZL (red) for undifferentiated *pbNANOS3*, *pbDAZL* and *pbMOCK* cells. Expression of PRKCSH was higher in *pbNANOS3* cells relative to *pbMOCK*, but no expression of OLFM2 was detected in either *pbNANOS3* or *pbMOCK* cells (not shown). Expression of IFITM3, ISYNA1 and PIDD1 was similar or lower in DAZL positive *pbDAZL* cells relative to DAZL negative *pbDAZL* cells or *pbMOCK* cells. No expression of CXCL5 was detected in either *pbDAZL* or *pbMOCK* cells (not shown). Scale bar indicates 100 μm.
(TIF)

S3 Fig Immunofluorescence staining for *in vitro* differentiation analysis. **A)** Undifferentiated *pbMOCK* cells were stained as a negative control for PLZF and GFRa1 (green), nuclei were counterstained for DAPI (blue). Scale bar indicates 200 μm. **B)** *pbMOCK*, *pbNANOS3* and *pbDAZL* cells differentiated for 14 days *in vitro* were stained for DDX4 (red), PRDM1 (red), SOX2 (green) and NANOG (red), nuclei were counterstained for DAPI (blue). Representative images are shown. Scale bar indicates 200 μm.
(TIF)

S4 Fig Histology of xenotransplanted testes. **A)** Teratoma formation was observed in at least one testis in each sample group, with representative tissue structures originating from ectoderm, endoderm and mesoderm. Scale bar 200 μm. **B)** Intratubular cell growth and teratoma-like differentiation was observed in all sample groups (arrows). Scale bar 200 μm. **C)** Varying degree of spermatogenesis was restored in busulfan treated mice, from Sertoli-cell only tubules to complete spermatogenesis. Restored spermatogenesis was assessed based on presence of round spermatids and/or mature spermatids. Scale bar 200 μm.
(TIF)

S1 File Methods. (DOCX)

S1 Table One-way ANOVA with Tukey’s multiple comparison test. Normalized Ct values to *GAPDH* and *RPLPO* were used for the analysis. Data represented as relative quantity in Fig 1B and 1E.
(DOCX)

S2 Table Differentially expressed genes by EdgeR analysis for mRNA sequencing data, genes with >1 FPKM. Related to data in Fig 2.
(DOCX)

S3 Table Two-way ANOVA with Bonferroni's multiple comparison test, comparison within cell line. Normalized Ct values to GAPDH and RPLPO were used for the analysis. Significantly different comparisons are shown. Data related to Fig 3A.
(DOCX)

S4 Table Summary of xenotransplantation assay. L = left testis, R = right testis, n/a = non applicable, + = few foci, ++ = several foci, +++ = dominating component. Data related to Fig 4.
(DOCX)
