# Supplementary figures and images for: Over Expression of NANOS3 and DAZL in Human Embryonic Stem Cells
Source: PLoS One. 2016 Oct 21;11(10):e0165268. doi: 10.1371/journal.pone.0165268 (PMC5074499; doi:10.1371/journal.pone.0165268)

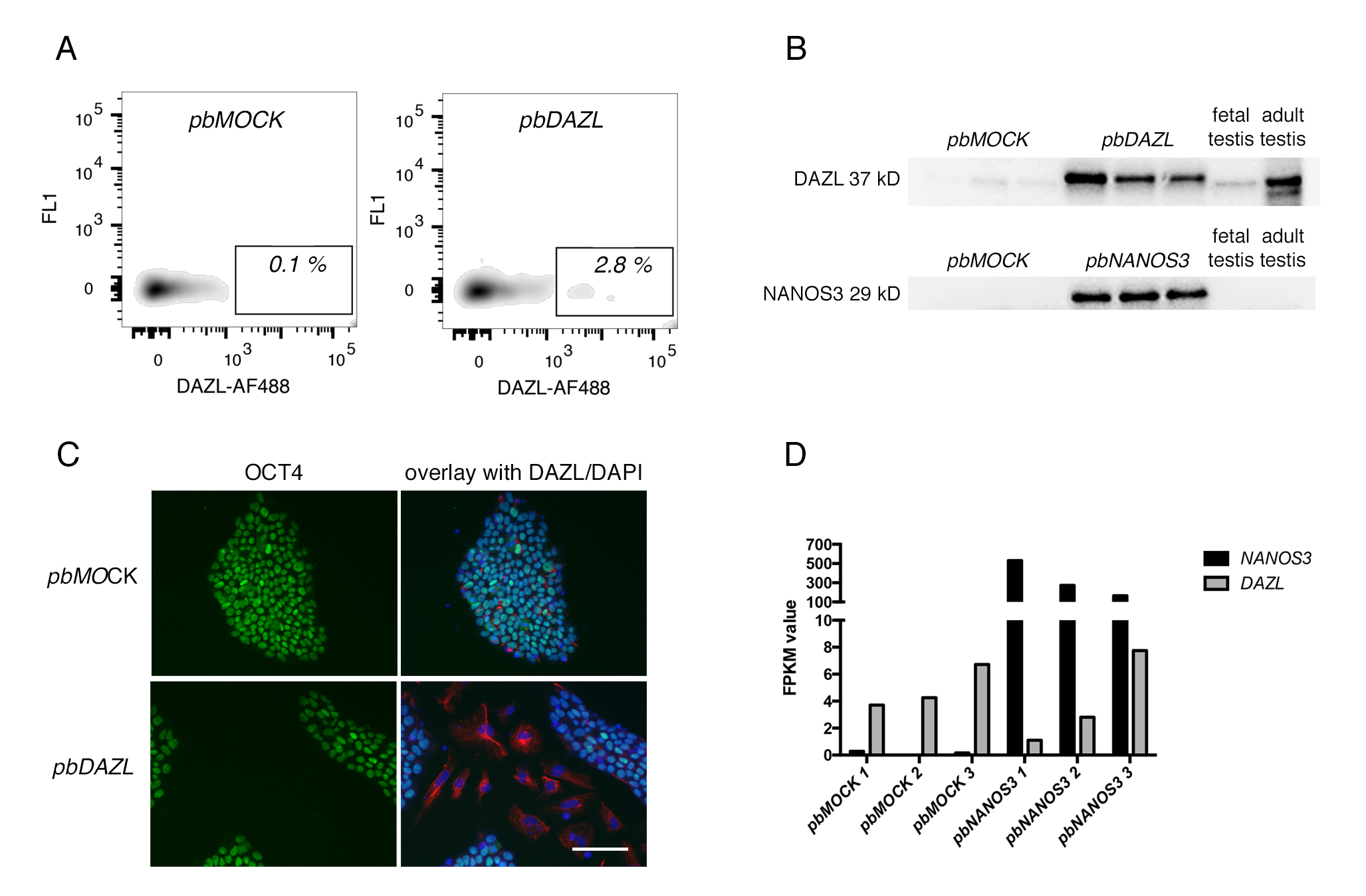

Supplement: S1 Fig — A) Flow cytometry analysis for quantification of DAZL positive cells in pbDAZL cultures. 2.8% of pbDAZL cells expressed DAZL protein. pbMOCK cells were used as negative control. B) Assessment of protein levels by Western blotting in over expressed cells relative to normal material obtained from the first trimester human fetal and adult testis samples. DAZL expression in pbDAZL cells was similar to adult testis sample, whereas no NANOS3 protein could be detected in either fetal or adult testis samples with this antibody. C) Immunofluorescence staining for OCT4 (green) and DAZL (red) to confirm lack of OCT4 expression in DAZL positive cells found outside the colonies. Scale bar indicates 100 μm. D) Gene expression analysis by mRNA sequencing. Expression of NANOS3 and DAZL are shown for biological replicates of pbMOCK and pbNANOS3 cells. The expression of DAZL is lower in pbNANOS3 samples with highest NANOS3 expression. Values are Fragments Per Kilobase Of Exon Per Million Fragments (FPKM). (TIF) [file pone.0165268.s001.tif]

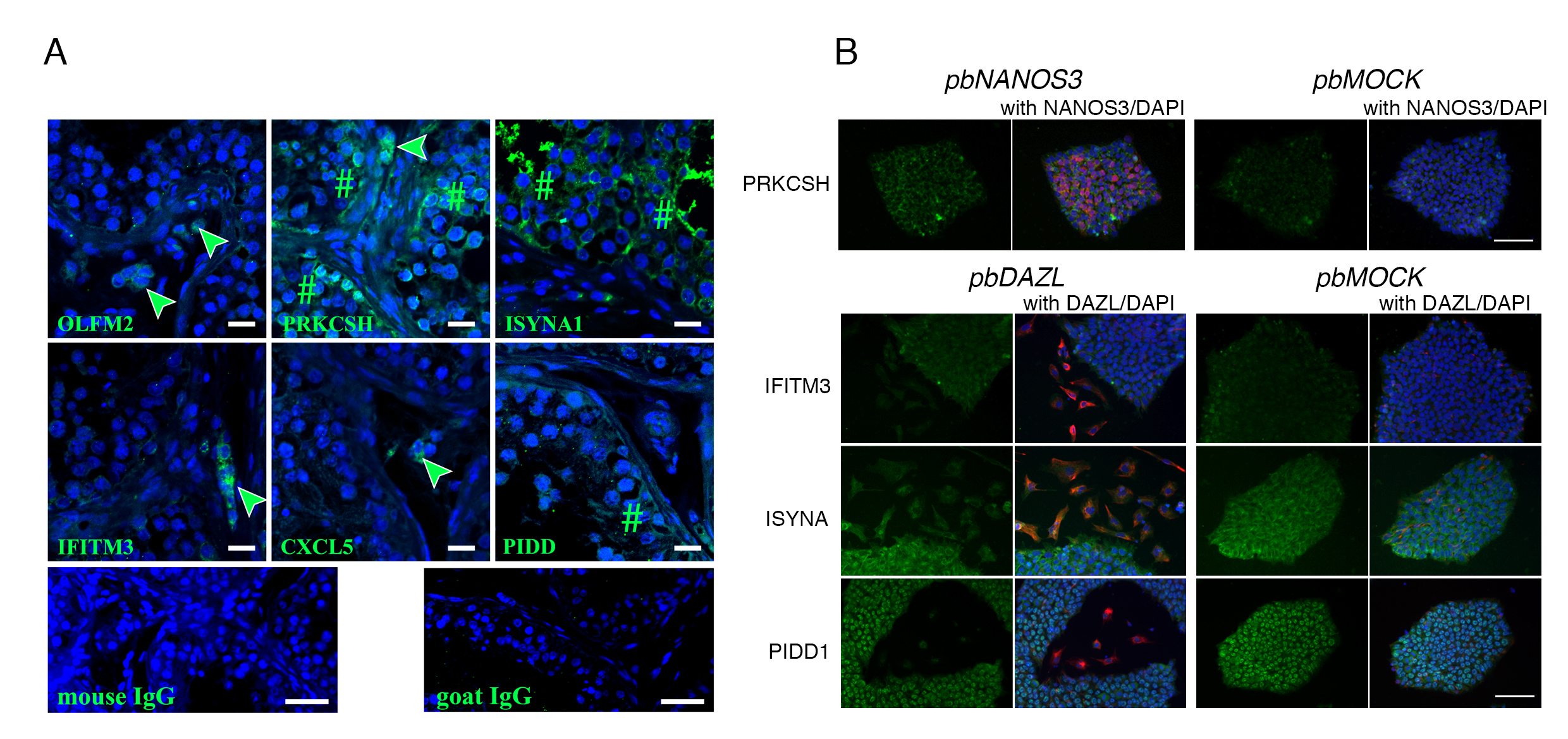

Supplement: S2 Fig — A) Human adult testis sections were immunostained for the selected markers (green) as a positive control. Arrowhead indicates Leydig cells and # indicates seminiferous tubular cells. Scale bar for the selected markers indicates 20 μm and for IgG controls 50 μm. B) Immunofluorescence staining of selected markers (green) together with NANOS3 (red) or DAZL (red) for undifferentiated pbNANOS3, pbDAZL and pbMOCK cells. Expression of PRKCSH was higher in pbNANOS3 cells relative to pbMOCK, but no expression of OLFM2 was detected in either pbNANOS3 or pbMOCK cells (not shown). Expression of IFITM3, ISYNA1 and PIDD1 was similar or lower in DAZL positive pbDAZL cells relative to DAZL negative pbDAZL cells or pbMOCK cells. No expression of CXCL5 was detected in either pbDAZL or pbMOCK cells (not shown). Scale bar indicates 100 μm. (TIF) [file pone.0165268.s002.tif]

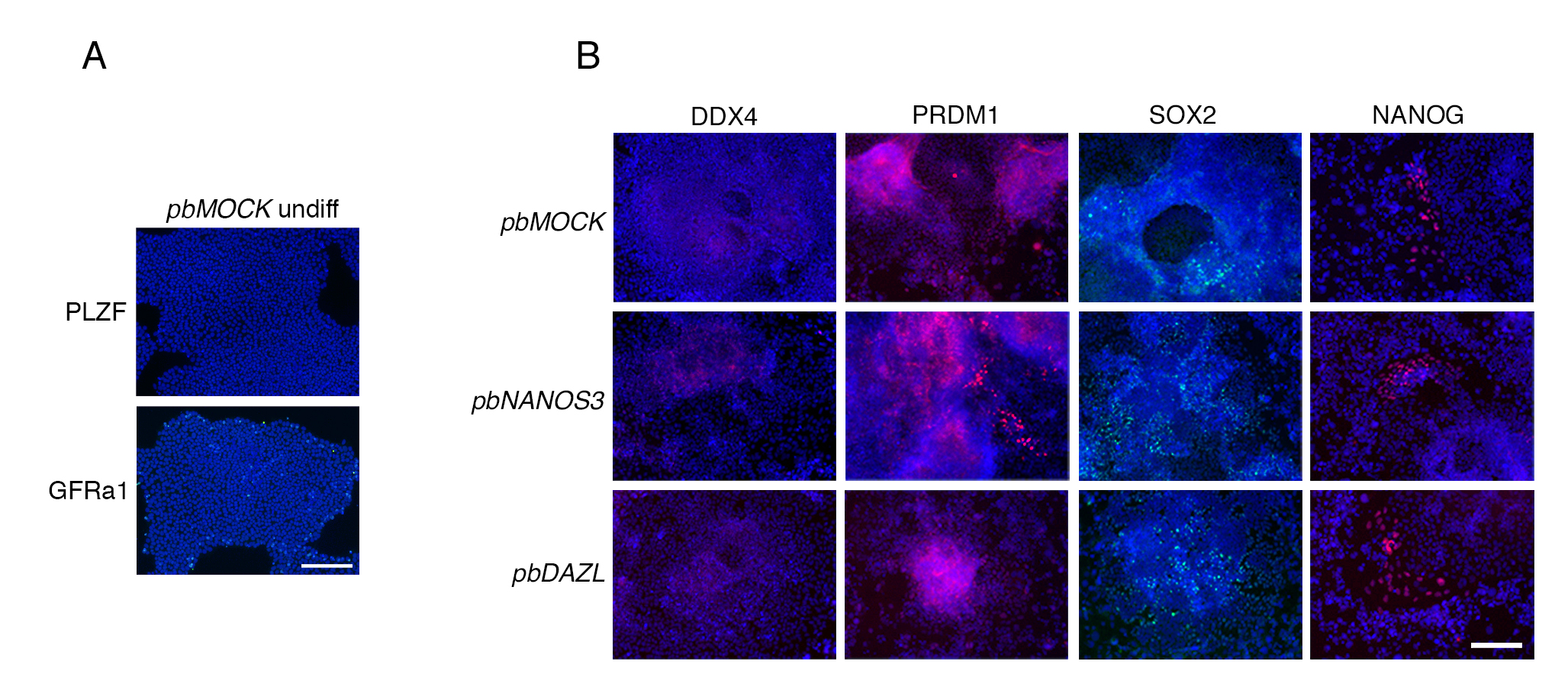

Supplement: S3 Fig — A) Undifferentiated pbMOCK cells were stained as a negative control for PLZF and GFRa1 (green), nuclei were counterstained for DAPI (blue). Scale bar indicates 200 μm. B) pbMOCK, pbNANOS3 and pbDAZL cells differentiated for 14 days in vitro were stained for DDX4 (red), PRDM1 (red), SOX2 (green) and NANOG (red), nuclei were counterstained for DAPI (blue). Representative images are shown. Scale bar indicates 200 μm. (TIF) [file pone.0165268.s003.tif]

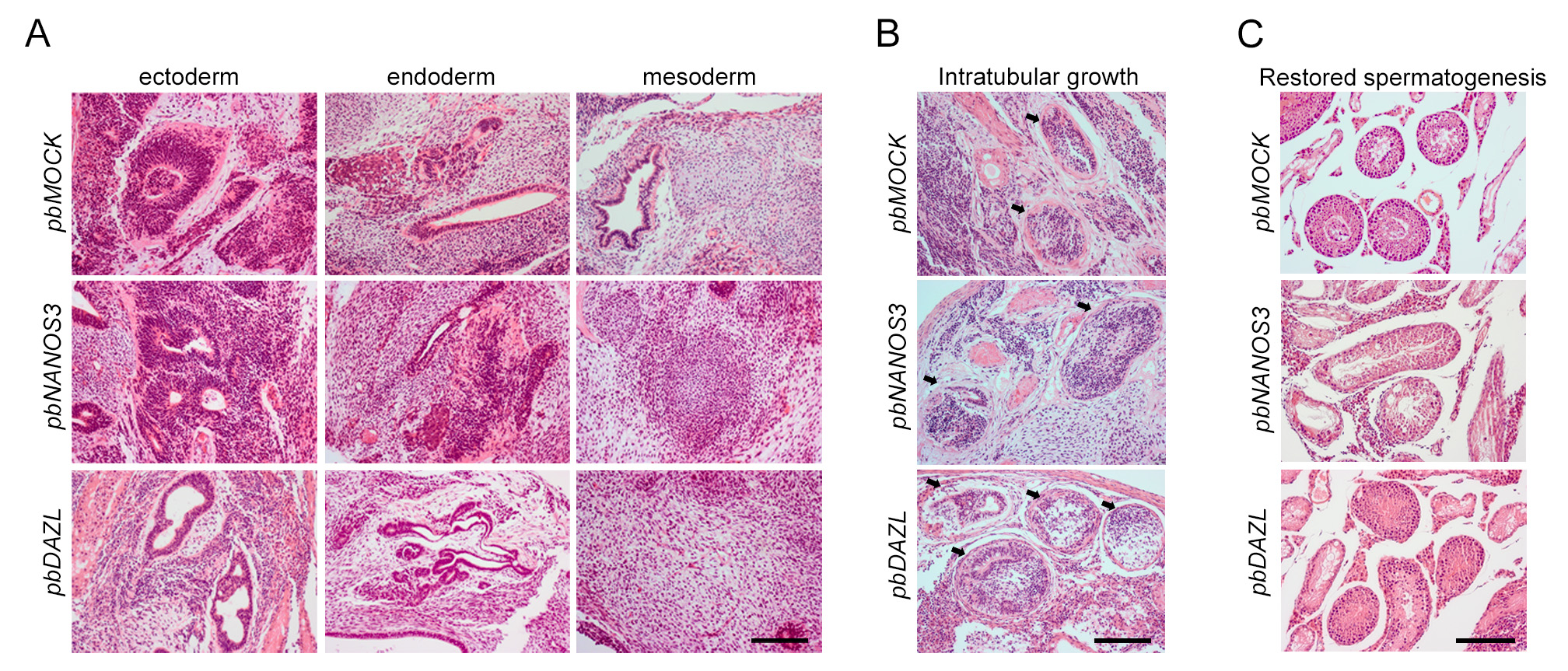

Supplement: S4 Fig — A) Teratoma formation was observed in at least one testis in each sample group, with representative tissue structures originating from ectoderm, endoderm and mesoderm. Scale bar 200 μm. B) Intratubular cell growth and teratoma-like differentiation was observed in all sample groups (arrows). Scale bar 200 μm. C) Varying degree of spermatogenesis was restored in busulfan treated mice, from Sertoli-cell only tubules to complete spermatogenesis. Restored spermatogenesis was assessed based on presence of round spermatids and/or mature spermatids. Scale bar 200 μm. (TIF) [file pone.0165268.s004.tif]
