# Supplementary material for: Over Expression of NANOS3 and DAZL in Human Embryonic Stem Cells
Source: PLoS One. 2016 Oct 21;11(10):e0165268. doi: 10.1371/journal.pone.0165268 (PMC5074499; doi:10.1371/journal.pone.0165268)
Supplement: S1 File — (DOCX) [file pone.0165268.s005.docx]

**Supporting Methods**

**Plasmid construction**

The *piggyBac* transposase expression vector pCX-IFP2 contained the *piggyBac* IFP2 gene driven by the CAG promoter. In brief, to construct the pCX-IFP2, BamHI fragment of pBSII-IFP2-orf [gift from Dr. Malcolm Fraser[1,2]] was subcloned into pCX-EGFP[3,4] digested with EcoRI (blunted) to replace the EGFP gene to obtain pCX-IFP2.

The *piggyBac* transposon vectors contained the open reading frame of the gene of interest, driven by the CAG promoter and followed by an IRES element and puromycin resistance gene, located between the 5’ and 3’ LTR regions. In brief, to construct the *piggyBac* transposon vectors, PB-MCS was generated by insertion of annealed oligos (sense: CTA GCG GCG GCG CGC CTC GAG GAT CCT TAA T, antisense: TAA GGA TCC TCG AGG CGC GCC G) into the NheI/PacI site of PB-CA-rtTA adv[5]. Followed by the insertion of SalI/BamHI fragment of pPyCAG-IP[6] into the XhoI/BamHI site of PB-MCS to obtain PB-CAG-IP. Gateway cassette rfA (Life Technologies) was inserted into the XhoI site of PB-CAG-IP to obtain PB-CAG-gw-IP. The open reading frames of *NANOS3* (NM_001098622.2, sense: CAC CAT GGG GAC CTT TGA CCT GTG GAC A, antisense: CTA GGT GGA CAT GGA GGG AGA GCA GGA G) and *DAZL* (NM_001190811.1, sense: CAC CAT GGC GGC TCC CTC GTG TGG, antisense: TCA AAC AGA TTT AAG CAT TGC CCG AC) were amplified by PCR using cDNA synthesized from human testis RNA (Clontech) as a template. The purified PCR products were cloned into pENTR/D-TOPO vectors (Life Technologies) and transferred to PB-CAG-gw-IP destination vector by the Gateway LR recombination system (Life Technologies) according to the manufacturer’s protocol.

**Q-PCR assays**

*GAPDH* (4333764F), *RPLPO* (4333761F), *DDX4* (Hs00987133_m1), *DAZL* (Hs00154706_m1), *OCT4* (Hs03005111_g1), *NANOG* (Hs02387400_g1), *SOX2* (Hs01053049_s1), *PLZF* (Hs00232313_m1), *PRDM1* (Hs00153357_m1), *PAX6* (Hs01088112_m1), and *NANOS3* (F: GGCGAAGACACAGGACACA, R: GGGCGAAGGCTCAGACTT, P: CCTCTGAAACCTGCTCCTC).

**Antibodies used for immunofluorescence staining of cells**

Primary antibodies used: 1:500 rabbit a-DDX4 (ab13840, Abcam), 1:2000 rabbit a-NANOS3 (ab70001, Abcam), 1:1000 rabbit a-DAZL (ab34139, Abcam), 1:300 mouse a-OCT4 (sc5279, Santa Cruz), 1:500 rabbit a-NANOG (ab21624, Abcam), 1:100 mouse a-PLZF (MAB2944, R&D Systems), 1:100 goat a-GFRa1 (AF714, R&D Systems), 1:100 rabbit a-PRDM1 (9115, Cell Signaling), 1:200 mouse a-SOX2 (MAB2018, R&D Systems), 1:100 goat a-OLFM2 (sc161140, Santa Cruz), 1:100 goat a-PIDD (sc32161, Santa Cruz), 1:100 mouse a-PRKCSH (sc374457, Santa Cruz), 1:100 mouse a-ISYNA1 (sc377245, Santa Cruz), 1:100 mouse a-FITM3 (sc100768, Santa Cruz), 1:100 mouse a-CXCL5 (sc377026, Santa Cruz). Secondary antibodies used: Alexa Fluor 488 Donkey a-mouse IgG, Alexa Fluor 594 Donkey a-rabbit IgG, Alexa Fluor 488 Donkey a-goat IgG, Alexa Fluor 488 Donkey a-rabbit IgG (all 1:1000, Life Technologies).

**Antibodies used for Western blotting**

Primary antibodies: 1:1000 DAZL (ab34139, Abcam), 1:2000 NANOS3 (ab70001, Abcam), 1:1000 GADPH (sc25778, Santa Cruz). Secondary antibodies: 1:3000 ECL a-rabbit IgG-HRP (GE Healthcare).

**Antibodies used for immunofluorescence staining of tissue sections**

Primary antibodies: 1:400 rabbit a-NUMA (ab84680, Abcam), 1:500 goat a-DDX4 (AF2030, R&D systems), 1:200 goat a-DPPA3 (ab78559, Abcam), 1:500 rabbit a-DAZL (ab34139, Abcam), 1:200 mouse a-UTF1 (mab4337, Millipore), 1:100 goat a-OLFM2 (sc161140, Santa Cruz), 1:100 goat a-PIDD (sc32161, Santa Cruz), 1:100 mouse a-PRKCSH (sc374457, Santa Cruz), 1:100 mouse a-ISYNA1 (sc377245, Santa Cruz), 1:100 mouse a-FITM3 (sc100768, Santa Cruz), 1:100 mouse a-CXCL5 (sc377026, Santa Cruz). Secondary antibodies: 1:200 chicken anti goat HRP (Santa Cruz), or chicken anti rabbit HRP (Santa Cruz), 1:1000 donkey anti mouse Alexa Fluor 488 (Jackson Immunoresearch), 1:1000 donkey anti goat Alexa Fluor 488 (Jackson Immunoresearch).

**Supporting references**

1. Cary LC, Goebel M, Corsaro BG, Wang HG, Rosen E, et al. (1989) Transposon mutagenesis of baculoviruses: analysis of Trichoplusia ni transposon IFP2 insertions within the FP-locus of nuclear polyhedrosis viruses. Virology 172: 156-169.

2. Fraser MJ, Cary L, Boonvisudhi K, Wang HG (1995) Assay for movement of Lepidopteran transposon IFP2 in insect cells using a baculovirus genome as a target DNA. Virology 211: 397-407.

3. Okabe M, Ikawa M, Kominami K, Nakanishi T, Nishimune Y (1997) 'Green mice' as a source of ubiquitous green cells. FEBS Lett 407: 313-319.

4. Niwa H, Yamamura K, Miyazaki J (1991) Efficient selection for high-expression transfectants with a novel eukaryotic vector. Gene 108: 193-199.

5. Woltjen K, Michael IP, Mohseni P, Desai R, Mileikovsky M, et al. (2009) piggyBac transposition reprograms fibroblasts to induced pluripotent stem cells. Nature 458: 766-770.

6. Niwa H, Burdon T, Chambers I, Smith A (1998) Self-renewal of pluripotent embryonic stem cells is mediated via activation of STAT3. Genes Dev 12: 2048-2060.
