# Supplementary material for: Over Expression of NANOS3 and DAZL in Human Embryonic Stem Cells
Source: PLoS One. 2016 Oct 21;11(10):e0165268. doi: 10.1371/journal.pone.0165268 (PMC5074499; doi:10.1371/journal.pone.0165268)
Supplement: S1 Table — Normalized Ct values to GAPDH and RPLPO were used for the analysis. Data represented as relative quantity in Fig 1B and 1E. (DOCX) [file pone.0165268.s006.docx]

**Supplementary Table 1: One-way ANOVA with Tukey’s multiple comparison test.** Normalized Ct values to *GAPDH* and *RPLPO* were used for the analysis. Data represented as relative quantity in Figure 1B and 1E.

| ***Gene*** | Comparison | Mean Diff. | 95% CI of diff. | Significant? | Summary | Adjusted P Value |
| --- | --- | --- | --- | --- | --- | --- |
|  | *pbMOCK* vs. *pbNANOS3* | 16.68 | 13.97 to 19.39 | Yes | **** | < 0.0001 |
| *NANOS3* | *pbMOCK* vs. *pbDAZL* | -0.7018 | -3.412 to 2.008 | No | ns | 0.8393 |
|  | *pbNANOS3* vs. *pbDAZ*L | -17.38 | -20.09 to -14.67 | Yes | **** | < 0.0001 |
| ***Gene*** | Comparison | Mean Diff. | 95% CI of diff. | Significant? | Summary | Adjusted P Value |
|  | *pbMOCK* vs. *pbNANOS3* | -1.152 | -3.241 to 0.9365 | No | ns | 0.3537 |
| *DAZL* | *pbMOCK* vs. *pbDAZL* | 4.608 | 2.519 to 6.697 | Yes | *** | 0.0005 |
|  | *pbNANOS3* vs. *pbDAZ*L | 5.761 | 3.672 to 7.849 | Yes | **** | < 0.0001 |
| ***Gene*** | Comparison | Mean Diff. | 95% CI of diff. | Significant? | Summary | Adjusted P Value |
|  | *pbMOCK* vs. *pbNANOS3* | -0.402 | -1.133 to 0.3292 | No | ns | 0.3563 |
| *OCT4* | *pbMOCK* vs. *pbDAZL* | 0.002198 | -0.7290 to 0.7334 | No | ns | > 0.9999 |
|  | *pbNANOS3* vs. *pbDAZ*L | 0.4042 | -0.3270 to 1.135 | No | ns | 0.3522 |
| ***Gene*** | Comparison | Mean Diff. | 95% CI of diff. | Significant? | Summary | Adjusted P Value |
|  | *pbMOCK* vs. *pbNANOS3* | 0.03558 | -1.100 to 1.171 | No | ns | 0.9996 |
| *NANOG* | *pbMOCK* vs. *pbDAZL* | 0.5484 | -0.5867 to 1.684 | No | ns | 0.4561 |
|  | *pbNANOS3* vs. *pbDAZ*L | 0.5128 | -0.6223 to 1.648 | No | ns | 0.5079 |
