# Supplementary material for: Over Expression of NANOS3 and DAZL in Human Embryonic Stem Cells
Source: PLoS One. 2016 Oct 21;11(10):e0165268. doi: 10.1371/journal.pone.0165268 (PMC5074499; doi:10.1371/journal.pone.0165268)
Supplement: S2 Table — Related to data in Fig 2. (DOCX) [file pone.0165268.s007.docx]

**Supplementary Table 2: Differentially expressed genes by EdgeR analysis for mRNA sequencing data, genes with >1 FPKM.** Related to data in Figure 2.

| Comparison | Gene | logFC | logCPM | LR | PValue | FDR |
| --- | --- | --- | --- | --- | --- | --- |
|  | NANOS3 | 11.2625374 | 4.950854515 | 511.5906947 | 2.86E-113 | 4.59E-109 |
| *pbNANOS3 vs* | OLFM2 | 1.016264539 | 5.89185503 | 26.07686123 | 3.28E-07 | 0.002635238 |
| *pbMOCK* | PRKCSH | 0.839660902 | 7.958506137 | 24.58939395 | 7.09E-07 | 0.00379866 |
|  | SLC38A5 | 0.747187141 | 5.468766979 | 19.6276563 | 9.41E-06 | 0.037789455 |
| Comparison | Gene | logFC | logCPM | LR | PValue | FDR |
|  | *DAZL* | 1.942104972 | 4.239373057 | 36.59466606 | 1.45E-09 | 2.34E-05 |
|  | *RP11-3B12.3* | 3.59243185 | -0.001213085 | 30.57226154 | 3.22E-08 | 0.000258357 |
|  | *COL3A1* | 5.407870974 | 1.291508504 | 27.89966558 | 1.28E-07 | 0.000684176 |
|  | *GDA* | 2.3968667 | 2.636699399 | 24.35932476 | 7.99E-07 | 0.003210344 |
|  | *CXCL5* | -1.332987852 | 3.903747043 | 23.6166098 | 1.18E-06 | 0.003777234 |
|  | *IGFBP7* | 2.793327759 | -0.188751347 | 22.77974912 | 1.82E-06 | 0.004863911 |
|  | *DKK2* | 3.612963452 | -1.078639722 | 20.92431995 | 4.78E-06 | 0.010666181 |
|  | *GABRP* | -1.437022356 | 3.285311011 | 20.72142133 | 5.31E-06 | 0.010666181 |
|  | *HIP1R* | 1.358122356 | 2.995962964 | 20.43542868 | 6.17E-06 | 0.011008723 |
|  | *ANXA3* | -1.234549567 | 5.522952348 | 19.69836053 | 9.07E-06 | 0.014566695 |
|  | *PRRX1* | 4.486027182 | -0.159918516 | 19.51082328 | 1.00E-05 | 0.014608206 |
|  | *PCDH18* | -0.709985205 | 6.397520612 | 19.28393155 | 1.13E-05 | 0.015080135 |
|  | *TUBB2A* | 1.29852331 | 6.143970825 | 18.34224494 | 1.85E-05 | 0.022657265 |
|  | *FAM35DP* | -2.068598534 | 1.432328983 | 18.21362458 | 1.97E-05 | 0.022657265 |
|  | *MAMDC2* | -0.733049604 | 5.199165364 | 16.95096341 | 3.84E-05 | 0.037215442 |
| *pbDAZL vs* | *ADAMTS7* | 1.050903775 | 4.942194295 | 16.94950804 | 3.84E-05 | 0.037215442 |
| *pbMOCK* | *FAM110C* | -0.97156368 | 4.251400425 | 16.90085341 | 3.94E-05 | 0.037215442 |
|  | *LCP1* | -1.380584477 | 5.815850961 | 16.75881907 | 4.24E-05 | 0.03787938 |
|  | *AGAP3* | 0.893025928 | 4.504298559 | 16.39097593 | 5.15E-05 | 0.03835287 |
|  | *ALOX12P2* | -1.40244998 | 1.951915881 | 16.34100279 | 5.29E-05 | 0.03835287 |
|  | *MSX2* | 3.170152066 | -0.119121256 | 16.16378186 | 5.81E-05 | 0.03835287 |
|  | *MIXL1* | 4.265562631 | 2.47616657 | 16.08463086 | 6.06E-05 | 0.03835287 |
|  | *ISYNA1* | 1.022356156 | 5.640195337 | 16.0502057 | 6.17E-05 | 0.03835287 |
|  | *EOMES* | 5.471473361 | 1.893898404 | 16.04412947 | 6.19E-05 | 0.03835287 |
|  | *PLEKHN1* | 2.058355545 | -0.23521693 | 15.9895824 | 6.37E-05 | 0.03835287 |
|  | *GRM4* | 1.113759764 | 4.692269109 | 15.96598608 | 6.45E-05 | 0.03835287 |
|  | *PPP1R16A* | 1.513589176 | 2.378855902 | 15.87727924 | 6.76E-05 | 0.03835287 |
|  | *LUM* | 6.996255271 | 0.285208504 | 15.77576012 | 7.13E-05 | 0.03835287 |
|  | *IFITM3* | 0.769171663 | 6.591061775 | 15.76488625 | 7.17E-05 | 0.03835287 |
|  | *PIDD1* | 1.555484045 | 3.633600247 | 15.71113726 | 7.38E-05 | 0.03835287 |
|  | *MDFI* | 1.209468003 | 3.679853723 | 15.70539833 | 7.40E-05 | 0.03835287 |
|  | *PWAR6* | -0.625343872 | 7.597318544 | 15.43438851 | 8.54E-05 | 0.042880662 |
|  | *LINC-ROR* | 1.600215216 | 3.409013153 | 15.34825046 | 8.94E-05 | 0.043468278 |
|  | *BMP7* | 1.138094511 | 3.892040556 | 15.28497978 | 9.24E-05 | 0.043468278 |
|  | *CACNA1H* | 1.00175423 | 4.525113395 | 15.21552127 | 9.59E-05 | 0.043468278 |
|  | *LRFN5* | 1.570736938 | 2.359270128 | 15.18617568 | 9.74E-05 | 0.043468278 |
|  | *JAG2* | 1.558029694 | 1.520308849 | 15.01735673 | 0.000106527 | 0.045291224 |
|  | *RPRM* | 1.449800512 | 1.51328151 | 15.00655987 | 0.000107138 | 0.045291224 |
