# Supplementary material for: Over Expression of NANOS3 and DAZL in Human Embryonic Stem Cells
Source: PLoS One. 2016 Oct 21;11(10):e0165268. doi: 10.1371/journal.pone.0165268 (PMC5074499; doi:10.1371/journal.pone.0165268)
Supplement: S3 Table — Normalized Ct values to GAPDH and RPLPO were used for the analysis. Significantly different comparisons are shown. Data related to Fig 3A. (DOCX) [file pone.0165268.s008.docx]

**Supplementary Table 3: Two-way ANOVA with Bonferroni's multiple comparison test, comparison within cell line.** Normalized Ct values to GAPDH and RPLPO were used for the analysis. Significantly different comparisons are shown. Data related to Figure 3A.

| ***DAZL*** | Comparison | Mean Diff. | 95% CI of diff. | Significant? | Summary | Adjusted P Value |
| --- | --- | --- | --- | --- | --- | --- |
|  | D0 vs. D5 | -2.065 | -3.716 to -0.4141 | Yes | ** | 0.0066 |
|  | D0 vs. D10 | -4.017 | -5.668 to -2.367 | Yes | **** | < 0.0001 |
|  | D0 vs. D14 | -8.693 | -10.34 to -7.042 | Yes | **** | < 0.0001 |
| pbMOCK | D5 vs. D10 | -1.952 | -3.603 to -0.3016 | Yes | * | 0.0117 |
|  | D5 vs. D14 | -6.628 | -8.279 to -4.977 | Yes | **** | < 0.0001 |
|  | D7 vs. D10 | -2.693 | -4.344 to -1.042 | Yes | *** | 0.0003 |
|  | D7 vs. D14 | -7.368 | -9.019 to -5.717 | Yes | **** | < 0.0001 |
|  | D10 vs. D14 | -4.675 | -6.326 to -3.024 | Yes | **** | < 0.0001 |
|  | D0 vs. D14 | -2.891 | -4.541 to -1.240 | Yes | **** | < 0.0001 |
| pbNANOS3 | D7 vs. D14 | -2.014 | -3.665 to -0.3636 | Yes | ** | 0.0085 |
|  | D10 vs. D14 | -1.74 | -3.390 to -0.08871 | Yes | * | 0.0329 |
| ***NANOG*** | Comparison | Mean Diff. | 95% CI of diff. | Significant? | Summary | Adjusted P Value |
|  | D0 vs. D7 | -4.104 | -6.061 to -2.148 | Yes | **** | < 0.0001 |
|  | D0 vs. D10 | -7.536 | -9.493 to -5.580 | Yes | **** | < 0.0001 |
|  | D0 vs. D14 | -9.205 | -11.16 to -7.249 | Yes | **** | < 0.0001 |
| pbMOCK | D5 vs. D7 | -2.3 | -4.257 to -0.3442 | Yes | * | 0.0123 |
|  | D5 vs. D10 | -5.733 | -7.689 to -3.776 | Yes | **** | < 0.0001 |
|  | D5 vs. D14 | -7.401 | -9.358 to -5.445 | Yes | **** | < 0.0001 |
|  | D7 vs. D10 | -3.432 | -5.388 to -1.476 | Yes | **** | < 0.0001 |
|  | D7 vs. D14 | -5.101 | -7.057 to -3.145 | Yes | **** | < 0.0001 |
|  | D0 vs. D10 | -3.727 | -5.684 to -1.771 | Yes | **** | < 0.0001 |
|  | D0 vs. D14 | -7.091 | -9.047 to -5.135 | Yes | **** | < 0.0001 |
| pbNANOS3 | D5 vs. D10 | -2.227 | -4.183 to -0.2708 | Yes | * | 0.0167 |
|  | D5 vs. D14 | -5.591 | -7.547 to -3.634 | Yes | **** | < 0.0001 |
|  | D7 vs. D10 | -2.126 | -4.083 to -0.1701 | Yes | * | 0.0253 |
|  | D7 vs. D14 | -5.49 | -7.446 to -3.534 | Yes | **** | < 0.0001 |
|  | D10 vs. D14 | -3.364 | -5.320 to -1.407 | Yes | *** | 0.0001 |
|  | D0 vs. D7 | -1.996 | -3.952 to -0.03967 | Yes | * | 0.0427 |
|  | D0 vs. D10 | -4.476 | -6.432 to -2.520 | Yes | **** | < 0.0001 |
|  | D0 vs. D14 | -7.704 | -9.660 to -5.748 | Yes | **** | < 0.0001 |
| pbDAZL | D5 vs. D10 | -3.337 | -5.294 to -1.381 | Yes | *** | 0.0001 |
|  | D5 vs. D14 | -6.566 | -8.522 to -4.610 | Yes | **** | < 0.0001 |
|  | D7 vs. D10 | -2.48 | -4.436 to -0.5237 | Yes | ** | 0.0058 |
|  | D7 vs. D14 | -5.708 | -7.664 to -3.752 | Yes | **** | < 0.0001 |
|  | D10 vs. D14 | -3.228 | -5.185 to -1.272 | Yes | *** | 0.0002 |
| ***NANOS3*** | Comparison | Mean Diff. | 95% CI of diff. | Significant? | Summary | Adjusted P Value |
|  | D0 vs. D5 | 2.736 | 0.5379 to 4.934 | Yes | ** | 0.007 |
|  | D0 vs. D7 | 2.395 | 0.1967 to 4.593 | Yes | * | 0.0248 |
|  | D0 vs. D10 | 4.114 | 1.916 to 6.312 | Yes | **** | < 0.0001 |
| pbMOCK | D0 vs. D14 | 5.737 | 3.539 to 7.935 | Yes | **** | < 0.0001 |
|  | D5 vs. D14 | 3.001 | 0.8027 to 5.199 | Yes | ** | 0.0025 |
|  | D7 vs. D14 | 3.342 | 1.144 to 5.540 | Yes | *** | 0.0007 |
|  | D0 vs. D5 | 2.623 | 0.4253 to 4.822 | Yes | * | 0.0107 |
|  | D0 vs. D7 | 2.27 | 0.07189 to 4.468 | Yes | * | 0.0388 |
|  | D0 vs. D10 | 3.501 | 1.303 to 5.699 | Yes | *** | 0.0004 |
| pbDAZL | D0 vs. D14 | 4.931 | 2.733 to 7.130 | Yes | **** | < 0.0001 |
|  | D5 vs. D14 | 2.308 | 0.1099 to 4.506 | Yes | * | 0.0339 |
|  | D7 vs. D14 | 2.661 | 0.4633 to 4.860 | Yes | ** | 0.0092 |
| ***OCT4*** | Comparison | Mean Diff. | 95% CI of diff. | Significant? | Summary | Adjusted P Value |
|  | D0 vs. D7 | -3.035 | -4.716 to -1.353 | Yes | **** | < 0.0001 |
|  | D0 vs. D10 | -7.255 | -8.936 to -5.573 | Yes | **** | < 0.0001 |
|  | D0 vs. D14 | -10.89 | -12.57 to -9.204 | Yes | **** | < 0.0001 |
| pbMOCK | D5 vs. D10 | -5.891 | -7.572 to -4.209 | Yes | **** | < 0.0001 |
|  | D5 vs. D14 | -9.522 | -11.20 to -7.840 | Yes | **** | < 0.0001 |
|  | D7 vs. D10 | -4.22 | -5.902 to -2.539 | Yes | **** | < 0.0001 |
|  | D7 vs. D14 | -7.851 | -9.533 to -6.170 | Yes | **** | < 0.0001 |
|  | D10 vs. D14 | -3.631 | -5.313 to -1.950 | Yes | **** | < 0.0001 |
|  | D0 vs. D10 | -2.762 | -4.443 to -1.080 | Yes | *** | 0.0002 |
|  | D0 vs. D14 | -5.969 | -7.650 to -4.287 | Yes | **** | < 0.0001 |
|  | D5 vs. D10 | -2.343 | -4.024 to -0.6611 | Yes | ** | 0.002 |
| pbNANOS3 | D5 vs. D14 | -5.55 | -7.231 to -3.868 | Yes | **** | < 0.0001 |
|  | D7 vs. D10 | -2.069 | -3.751 to -0.3876 | Yes | ** | 0.0079 |
|  | D7 vs. D14 | -5.276 | -6.958 to -3.595 | Yes | **** | < 0.0001 |
|  | D10 vs. D14 | -3.207 | -4.889 to -1.526 | Yes | **** | < 0.0001 |
|  | D0 vs. D10 | -4.557 | -6.239 to -2.876 | Yes | **** | < 0.0001 |
|  | D0 vs. D14 | -8.19 | -9.872 to -6.508 | Yes | **** | < 0.0001 |
| pbDAZL | D5 vs. D10 | -3.973 | -5.655 to -2.292 | Yes | **** | < 0.0001 |
|  | D5 vs. D14 | -7.606 | -9.288 to -5.925 | Yes | **** | < 0.0001 |
|  | D7 vs. D10 | -3.521 | -5.202 to -1.839 | Yes | **** | < 0.0001 |
|  | D7 vs. D14 | -7.153 | -8.835 to -5.472 | Yes | **** | < 0.0001 |
|  | D10 vs. D14 | -3.633 | -5.314 to -1.951 | Yes | **** | < 0.0001 |
| ***PLZF*** | Comparison | Mean Diff. | 95% CI of diff. | Significant? | Summary | Adjusted P Value |
|  | D0 vs. D7 | 7.216 | 5.257 to 9.176 | Yes | **** | < 0.0001 |
|  | D0 vs. D10 | 9.742 | 7.782 to 11.70 | Yes | **** | < 0.0001 |
|  | D0 vs. D14 | 8.458 | 6.498 to 10.42 | Yes | **** | < 0.0001 |
| pbMOCK | D5 vs. D7 | 5.6 | 3.641 to 7.560 | Yes | **** | < 0.0001 |
|  | D5 vs. D10 | 8.126 | 6.167 to 10.09 | Yes | **** | < 0.0001 |
|  | D5 vs. D14 | 6.842 | 4.882 to 8.801 | Yes | **** | < 0.0001 |
|  | D7 vs. D10 | 2.526 | 0.5663 to 4.485 | Yes | ** | 0.0048 |
|  | D0 vs. D7 | 5.389 | 3.430 to 7.349 | Yes | **** | < 0.0001 |
|  | D0 vs. D10 | 8.675 | 6.715 to 10.63 | Yes | **** | < 0.0001 |
|  | D0 vs. D14 | 9.381 | 7.421 to 11.34 | Yes | **** | < 0.0001 |
| pbNANOS3 | D5 vs. D7 | 4.84 | 2.881 to 6.800 | Yes | **** | < 0.0001 |
|  | D5 vs. D10 | 8.126 | 6.166 to 10.09 | Yes | **** | < 0.0001 |
|  | D5 vs. D14 | 8.832 | 6.872 to 10.79 | Yes | **** | < 0.0001 |
|  | D7 vs. D10 | 3.285 | 1.326 to 5.245 | Yes | *** | 0.0002 |
|  | D7 vs. D14 | 3.991 | 2.032 to 5.951 | Yes | **** | < 0.0001 |
|  | D0 vs. D7 | 7.25 | 5.290 to 9.209 | Yes | **** | < 0.0001 |
|  | D0 vs. D10 | 10.19 | 8.226 to 12.15 | Yes | **** | < 0.0001 |
|  | D0 vs. D14 | 9.404 | 7.445 to 11.36 | Yes | **** | < 0.0001 |
| pbDAZL | D5 vs. D7 | 5.952 | 3.992 to 7.911 | Yes | **** | < 0.0001 |
|  | D5 vs. D10 | 8.888 | 6.928 to 10.85 | Yes | **** | < 0.0001 |
|  | D5 vs. D14 | 8.106 | 6.147 to 10.07 | Yes | **** | < 0.0001 |
|  | D7 vs. D10 | 2.936 | 0.9765 to 4.895 | Yes | *** | 0.0008 |
|  | D7 vs. D14 | 2.154 | 0.1949 to 4.114 | Yes | * | 0.0229 |
| ***PRDM1*** | Comparison | Mean Diff. | 95% CI of diff. | Significant? | Summary | Adjusted P Value |
|  | D0 vs. D5 | 3.825 | 2.392 to 5.257 | Yes | **** | < 0.0001 |
|  | D0 vs. D7 | 4.682 | 3.250 to 6.115 | Yes | **** | < 0.0001 |
| pbMOCK | D0 vs. D10 | 3.989 | 2.556 to 5.421 | Yes | **** | < 0.0001 |
|  | D0 vs. D14 | 2.41 | 0.9776 to 3.843 | Yes | *** | 0.0002 |
|  | D7 vs. D14 | -2.272 | -3.704 to -0.8395 | Yes | *** | 0.0004 |
|  | D10 vs. D14 | -1.579 | -3.011 to -0.1464 | Yes | * | 0.0224 |
|  | D0 vs. D5 | 3.224 | 1.791 to 4.656 | Yes | **** | < 0.0001 |
| pbNANOS3 | D0 vs. D7 | 4.684 | 3.251 to 6.116 | Yes | **** | < 0.0001 |
|  | D0 vs. D10 | 4.464 | 3.031 to 5.896 | Yes | **** | < 0.0001 |
|  | D0 vs. D14 | 3.911 | 2.479 to 5.344 | Yes | **** | < 0.0001 |
|  | D5 vs. D7 | 1.46 | 0.02732 to 2.892 | Yes | * | 0.0431 |
|  | D0 vs. D5 | 4.304 | 2.872 to 5.737 | Yes | **** | < 0.0001 |
|  | D0 vs. D7 | 5.274 | 3.842 to 6.707 | Yes | **** | < 0.0001 |
| pbDAZL | D0 vs. D10 | 4.861 | 3.428 to 6.293 | Yes | **** | < 0.0001 |
|  | D0 vs. D14 | 3.966 | 2.534 to 5.399 | Yes | **** | < 0.0001 |
| ***SOX2*** | Comparison | Mean Diff. | 95% CI of diff. | Significant? | Summary | Adjusted P Value |
| pbMOCK | D5 vs. D14 | -1.516 | -2.897 to -0.1354 | Yes | * | 0.0231 |
|  | D7 vs. D14 | -1.867 | -3.247 to -0.4858 | Yes | ** | 0.0028 |
| pbDAZL | D5 vs. D14 | -1.423 | -2.804 to -0.04239 | Yes | * | 0.0394 |
|  | D7 vs. D14 | -1.511 | -2.892 to -0.1301 | Yes | * | 0.0238 |
|  | D10 vs. D14 | -1.471 | -2.852 to -0.09042 | Yes | * | 0.03 |
| ***PAX6*** | Comparison | Mean Diff. | 95% CI of diff. | Significant? | Summary | Adjusted P Value |
|  | D0 vs. D5 | 6.939 | 5.089 to 8.788 | Yes | **** | < 0.0001 |
|  | D0 vs. D7 | 10.53 | 8.685 to 12.38 | Yes | **** | < 0.0001 |
|  | D0 vs. D10 | 12.02 | 10.17 to 13.87 | Yes | **** | < 0.0001 |
|  | D0 vs. D14 | 11.62 | 9.775 to 13.47 | Yes | **** | < 0.0001 |
| pbMOCK | D5 vs. D7 | 3.596 | 1.747 to 5.446 | Yes | **** | < 0.0001 |
|  | D5 vs. D10 | 5.082 | 3.233 to 6.931 | Yes | **** | < 0.0001 |
|  | D5 vs. D14 | 4.685 | 2.836 to 6.535 | Yes | **** | < 0.0001 |
|  | D0 vs. D5 | 5.012 | 3.163 to 6.861 | Yes | **** | < 0.0001 |
|  | D0 vs. D7 | 7.963 | 6.113 to 9.812 | Yes | **** | < 0.0001 |
|  | D0 vs. D10 | 10.46 | 8.614 to 12.31 | Yes | **** | < 0.0001 |
|  | D0 vs. D14 | 10.7 | 8.854 to 12.55 | Yes | **** | < 0.0001 |
| pbNANOS3 | D5 vs. D7 | 2.951 | 1.101 to 4.800 | Yes | *** | 0.0003 |
|  | D5 vs. D10 | 5.452 | 3.602 to 7.301 | Yes | **** | < 0.0001 |
|  | D5 vs. D14 | 5.691 | 3.842 to 7.541 | Yes | **** | < 0.0001 |
|  | D7 vs. D10 | 2.501 | 0.6515 to 4.350 | Yes | ** | 0.0028 |
|  | D7 vs. D14 | 2.741 | 0.8913 to 4.590 | Yes | *** | 0.0009 |
|  | D0 vs. D5 | 7.3 | 5.451 to 9.149 | Yes | **** | < 0.0001 |
|  | D0 vs. D7 | 10.89 | 9.038 to 12.74 | Yes | **** | < 0.0001 |
|  | D0 vs. D10 | 12.82 | 10.97 to 14.67 | Yes | **** | < 0.0001 |
|  | D0 vs. D14 | 12.87 | 11.02 to 14.72 | Yes | **** | < 0.0001 |
| pbDAZL | D5 vs. D7 | 3.588 | 1.738 to 5.437 | Yes | **** | < 0.0001 |
|  | D5 vs. D10 | 5.517 | 3.667 to 7.366 | Yes | **** | < 0.0001 |
|  | D5 vs. D14 | 5.57 | 3.721 to 7.419 | Yes | **** | < 0.0001 |
|  | D7 vs. D10 | 1.929 | 0.07975 to 3.779 | Yes | * | 0.0357 |
|  | D7 vs. D14 | 1.982 | 0.1329 to 3.832 | Yes | * | 0.0285 |
