# Supplementary material for: Over Expression of NANOS3 and DAZL in Human Embryonic Stem Cells
Source: PLoS One. 2016 Oct 21;11(10):e0165268. doi: 10.1371/journal.pone.0165268 (PMC5074499; doi:10.1371/journal.pone.0165268)
Supplement: S4 Table — L = left testis, R = right testis, n/a = non applicable, + = few foci, ++ = several foci, +++ = dominating component. Data related to Fig 4. (DOCX) [file pone.0165268.s009.docx]

**Supplementary Table 4*:* Summary of xenotransplantation assay.** L = left testis, R = right testis, n/a = non applicable, + = few foci, ++ = several foci, +++ = dominating component. Data related to Figure 4.

| **Cell line** | **Mouse** | | **Injected cell number** | **Transplantation time** | **Xenograft weight (mg)** | **Outcome** | **Analysis** | **Whole mount results** | | **Sectioning results** | | | |
| --- | --- | --- | --- | --- | --- | --- | --- | --- | --- | --- | --- | --- | --- |
|  |  |  |  |  |  |  |  | **Colonies** | **Cells in colonies** | **NuMA+ tubules (%)** | **Teratoma-like** | **Restored Spermatogenesis** | **Necrosis** |
| *pbMOCK* | 4185 | R | 4.24E+05 | 5 weeks | 24.88 | Soft | Whole mount | 0 | n/a |  |  |  |  |
|  |  | L | 1.49E+06 |  | 49.44 | Soft | Sectioning |  |  |  |  |  |  |
|  | 4186 | R | 0.00E+00 | 7 weeks | 22.86 | Soft | Whole mount | 0 | n/a |  |  |  |  |
|  |  | L | 1.49E+06 |  | 83.84 | Hard | Sectioning |  |  | 0.2 | Yes | + | ++ |
|  | 4187 | R | 1.27E+06 | 4 weeks | n/a | No testis found | n/a |  |  |  |  |  |  |
|  |  | L | 1.27E+06 |  | n/a |  | n/a |  |  |  |  |  |  |
|  | 4188 | R | 1.27E+06 | 1 week | 24.26 | Soft | Whole mount | 0 | n/a |  |  |  |  |
|  |  | L | 1.27E+06 |  | 24.78 | Soft | Sectioning |  |  |  |  |  |  |
|  | 4189 | R | 1.49E+06 | 8 weeks | 114.21 | Hard | Sectioning |  |  | 12.7 | Yes | No | + |
|  |  | L | 1.49E+06 |  | 111.86 | Hard | Sectioning |  |  | 4.4 | No | ++ | ++ |
|  | 4190 | R | 1.49E+06 | 8 weeks | 48.03 | Soft | Whole mount | 0 | n/a |  |  |  |  |
|  |  | L | 1.49E+06 |  | 45.47 | Soft | Sectioning |  |  | 0.0 | No | +++ | No |
|  | 4191 | R | 1.49E+06 | 8 weeks | 245.92 | Tumor | Sectioning |  |  |  | No | + | ++ |
|  |  | L | 1.27E+06 |  | 248.93 | Tumor | Sectioning |  |  |  | Yes | + | + |
| *pbNANOS3* | 4201 | R | 1.24E+06 | 8 weeks | 126.77 | Tumor | Sectioning |  |  |  |  |  |  |
|  |  | L | 1.45E+06 |  | 111.97 | Tumor | Sectioning |  |  | 20.0 | Yes | No | ++ |
|  | 4202 | R | 1.45E+06 | 8 weeks | 19.36 | Shrunken | Sectioning |  |  |  |  |  |  |
|  |  | L | 1.24E+06 |  | 124.52 | Tumor | Sectioning |  |  |  |  |  |  |
|  | 4203 | R | 1.45E+06 | 8 weeks | 255.64 | Tumor | Sectioning |  |  |  | No | No | +++ |
|  |  | L | 1.45E+06 |  | 476.89 | Tumor | Sectioning |  |  |  |  |  |  |
|  | 4204 | R | 1.45E+06 | 8 weeks | 102.14 | Tumor | Sectioning |  |  | 0.5 | No | No | +++ |
|  |  | L | 1.45E+06 |  | 93.19 | Tumor | Sectioning |  |  |  |  |  |  |
|  | 4205 | R | 1.45E+06 | 8 weeks | 411.59 | Tumor | Sectioning |  |  |  |  |  |  |
|  |  | L | 1.45E+06 |  | 315.61 | Tumor | Sectioning |  |  |  | Yes | No | No |
|  | 4206 | R | 1.45E+06 | 8 weeks | 75.03 | Tumor | Sectioning |  |  | 0.5 | Yes | + | No |
|  |  | L | 1.45E+06 |  | 52.93 | Tumor | Sectioning |  |  |  |  |  |  |
|  | 4207 | R | 1.45E+06 | 5 weeks | 75.03 | Soft | Whole mount | 1 | clump |  |  |  |  |
|  |  | L | 1.45E+06 |  | 52.93 | Soft | Sectioning |  |  | 5.5 | No | + | No |
| *pbDAZL* | 4154 | R | 1.42E+06 | 8 weeks | 60.90 | Partial soft | Sectioning |  |  |  |  |  |  |
|  |  | L | 1.42E+06 |  | 149.45 | Soft - Tumor | Sectioning |  |  | 4.9 | Yes | No | No |
|  | 4155 | R | 1.42E+06 | 8 weeks | 30.10 | Soft | Whole mount | 0 | n/a |  |  |  |  |
|  |  | L | 1.22E+06 |  | 40.22 | Soft | Sectioning |  |  | 0.8 | Yes | No | No |
|  | 4156 | R | 1.42E+06 | 8 weeks | 67.89 | Partial soft | Whole mount | 6 | 18, 9, 5, 8, 13, 12 |  |  |  |  |
|  |  | L | 1.42E+06 |  | 90.74 | Hard - Tumor | Sectioning |  |  | 5.3 | Yes | + | ++ |
|  | 4157 | R | 1.42E+06 | 8 weeks | 34.34 | Soft | Whole mount | 0 | n/a |  |  |  |  |
|  |  | L | 1.42E+06 |  | 37.29 | Soft | Sectioning |  |  | 2.5 | Yes | + | No |
|  | 4158 | R | 1.01E+06 | 8 weeks | 92.92 | Hard - Tumor | Sectioning |  |  |  |  |  |  |
|  |  | L | 1.01E+06 |  | 100.09 | Hard - Tumor | Sectioning |  |  |  |  |  |  |
|  | 4159 | R | 1.42E+06 | 8 weeks | 85.54 | Hard - Tumor | Sectioning |  |  |  |  |  |  |
|  |  | L | 1.42E+06 |  | 45.83 | Soft | Sectioning |  |  |  |  |  |  |
|  | 4160 | R | 1.42E+06 | 8 weeks | 97.89 | Hard - Tumor | Sectioning |  |  | 0.7 | Yes | No | + |
|  |  | L | 1.42E+06 |  | 74.81 | Hard - Tumor | Sectioning |  |  | 0.0 | No | + | No |
